# Interventions for Neglected Diseases Caused by Kinetoplastid Parasites: A One Health Approach to Drug Discovery, Development, and Deployment

**DOI:** 10.3390/ph18091415

**Published:** 2025-09-19

**Authors:** Godwin U. Ebiloma, Amani Alhejeli, Harry P. de Koning

**Affiliations:** 1 School of Science, Engineering & Environment, University of Salford, Manchester M5 4NT, UK; 2 Biology Department, Darb University College, Jazan 45142, Saudi Arabia; amalhujaili@hotmail.com; 3 School of Infection and Immunity, University of Glasgow, Glasgow G12 8TA, UK; harry.de-koning@glasgow.ac.uk

**Keywords:** kinetoplastid parasites, Chagas disease, sleeping sickness, leishmaniasis, One Health, zoonoses, drug discovery, vector-borne diseases, neglected tropical diseases (NTDs), drug resistance

## Abstract

Kinetoplastids are protozoa that possess a unique organelle called a kinetoplast. These include the parasites *Trypanosoma cruzi*, *T. brucei* and related *African trypanosomes*, and *Leishmania* spp. These parasites cause a variety of neglected tropical diseases in humans and livestock, with devastating consequences. In the absence of any vaccine, pharmaceutical interventions are the mainstay of control, but these have historically been underfunded, fragmented, and inadequately aligned with the complex zoonotic and ecological realities of the parasites’ transmission dynamics. In this review, the landscape of current and emerging drugs for treating leishmaniasis, Chagas disease, and African trypanosomiasis is critically evaluated across both veterinary and human contexts. It examines the challenges of legacy compounds, the pharmacological shortcomings in multi-host, multi-tropic and multi-stage disease systems, and the gaps in veterinary therapeutics, specifically for African animal trypanosomiasis and canine leishmaniasis but also the animal reservoir of *T. cruzi*. Emphasis is placed on pharmacokinetic divergence between species, the accompanying risks with the use of off-label human drugs in animals, and the ecological effects of environmental drug exposure. We propose a far-reaching One Health framework for pharmaceutical research and development, promoting dual-indication co-development, ecological pharmacology, regulatory harmonisation, and integrated delivery systems. In this context, we argue that the drug development pipeline must be rationalised as a transdisciplinary and ecologically embedded process, able to interrupt parasite transmission to human, animal, and vector interfaces. Our findings reveal that we can bridge age-old therapeutic gaps, advance towards sustainable control, and eventually eliminate the neglected diseases caused by kinetoplastid protozoan parasites by aligning pharmaceutical innovation with One Health principles. This article aims to promote future research and development of innovative drugs that are sustainable under the One Health framework.

## 1. Introduction

Kinetoplastid diseases comprise a subgroup of the neglected tropical diseases (NTDs) and are caused by flagellated protozoan parasites of the class Kinetoplastida. These diseases—human African trypanosomiasis (HAT), Chagas disease, and leishmaniasis—are caused by the *Trypanosoma brucei* subspecies *T. b. gambiense* and *T. b. rhodesiense*, by *Trypanosoma cruzi*, and by approximately 20 different *Leishmania* spp., respectively [1]. Leishmaniasis appears in cutaneous, visceral and mucocutaneous forms depending on parasite species host-related factors such as immune status, further complicating treatment standardisation.

HAT is geographically limited to the habitat of the tsetse fly vector in sub-Saharan Africa [2] but some forms of animal African trypanosomiasis (AAT) are not dependent on tsetse for transmission and have spread to much of the rest of the globe [3,4,5]. Chagas disease is endemic to South and Central America but transmission via the triatomine vector has now spread through Mexico and into the southern United States [6]. In addition, congenital and iatrogenic transmission, e.g., via blood transfusion or organ transplant, are possible globally due to migration of symptomatic and asymptomatic carriers [7,8]. Leishmaniasis is transmitted by phlebotomine sandflies and endemic in 90 countries; foci are the Indian subcontinent, the Middle East and Sudan, and the Amazon region of South America, but it is still spreading, including northwards spread from southern Europe as the sandfly habitat expands as a result of climate change [9,10].

The kinetoplastid diseases affect people as well as domestic and wild animals, and are most endemic in sub-Saharan Africa, Latin America, the Middle East, and South Asia, where they mainly impact populations already living in poverty with poor access to quality healthcare, basic infrastructure, and vector control [2,11]. The burden of kinetoplastid infections goes beyond mortality and morbidity to include profound socioeconomic effects, including reduced productivity, social stigmatisation, and impediments to development [12,13,14]. Although these diseases are justly categorised as “neglected,” the medical importance of kinetoplastid diseases is substantial, with millions at risk and hundreds of thousands of new cases occurring annually in the endemic regions [15].

Pharmaceutical interventions for kinetoplastid diseases have for decades now trailed far behind those for most other infectious diseases, even though drugs against sleeping sickness, in particular, were first developed in the early 20th century [16]. As a result, the currently used old drugs, such as benznidazole, pentavalent antimonials, melarsoprol, eflornithine and nifurtimox, are severely challenged by issues including severe toxicity, low efficacy, drug counterfeiting, resistance, and prolonged treatment regimens [17,18]. Recently, the first oral drugs for leishmaniasis (miltefosine) and now sleeping sickness (fexinidazole) have been introduced and more antikinetoplastid agents are in development (acoziborole and DNDi-0690); while highly welcome, these interventions are not universally accessible, and their long-term impact on disease transmission dynamics remains uncertain [19,20,21]. However, it could be argued that the most important issue of all is that, to date, the pharmaceutical strategy has focused narrowly on treating infected humans and overlooked the broader zoonotic and ecological contexts in which these diseases are transmitted, leaving the animal reservoir mostly untreated and uncontrolled [22].

Kinetoplastid parasites are vector-borne pathogens with a complex life cycle involving both vertebrate and invertebrate hosts. Dogs, rodents, and wild mammals are reservoirs for *T. cruzi* and *Leishmania* spp., which are transmitted by infected blood-sucking triatomine (‘kissing bugs’) and sandflies, respectively [23,24,25]. Tsetse flies (*Glossina* spp.) mediate the transmission of African trypanosomes including *T. b. gambiense* and *T. b. rhodesiense* among humans, livestock, and wildlife, as well as the transmission of *T. congolense*, *T. evansi* and *T. vivax*, among others, which rarely infect humans [5,26]. This interconnected transmission ecology makes human-centric interventions inadequate for sustained disease control. Pharmaceutical strategies, though valuable, are often conducted independently of public health campaigns and vector control programs, and drug development efforts explicitly overlook animal reservoirs or vector dynamics [11,27]. Such a fragmented strategy neglects opportunities for efficiency and synergy.

The One Health framework provides a compelling alternative. By recognising the interconnection of human, animal, and environment, the One Health approach to drug discovery encourages a robust, integrated, cross-sectoral strategy to sustainable disease management [28,29,30]. For kinetoplastid diseases, this requires reconsidering pharmaceutical interventions to include veterinary therapeutics, environmental stewardship, and collective surveillance systems—a holistic approach to observe, monitor and treat the disease in its zoonotic totality. It recognises the need to design effective drugs for both human and animal usage (whether the same or separate drugs). It also includes deploying them in ways that take into account the ecological context, and multisectoral monitoring platforms [31].

In this review, the pharmaceutical landscape for kinetoplastid diseases is examined through the lens of One Health. A critical appraisal of current and emerging pharmaceutical interventions is provided, their compatibility with integrated control strategies is assessed, and opportunities for more effective and sustainable approaches are identified. This review includes a compendium of a more-than-one-hundred-year-old trend of registered drugs for treating human and animal diseases caused by kinetoplastid protozoan parasites, including their mechanism of action. It concludes with actionable suggestions for aligning drug development with One Health principles, emphasising the need for equitable access, interdisciplinary collaboration, and environmental responsibility.

## 2. Kinetoplastid Parasites and the One Health Paradigm

Kinetoplastid parasites, such as the trypanosomatids, belong to the order Kinetoplastida and possess a unique organelle called the kinetoplast—a specialised organelle within the mitochondrion that harbours a complex and unusual mitochondrial DNA (kDNA), which is essential for parasite survival and life cycle [32]. These transmission cycles are essentially zoonotic, involving ecologically complex interactions between vectors and multiple vertebrate hosts, including domestic animals, livestock, and wildlife [33], and this can be understood through the critical lens of the One Health framework.

The One Health approach underscores the interrelationship between human, animal, and environmental health. In the context of kinetoplastid diseases, it involves a pronounced consideration for kinetoplastid parasites circulating in multispecies networks across rural, urban, and sylvatic settings. Within these transmission ecosystems, interventions focusing exclusively on human cases are effectively limited in their scope and sustainability [34].

For example, in the case of visceral leishmaniasis caused by *L. infantum*, domestic dogs are not only victims of the infection but also serve as the primary reservoirs, facilitating transmission to humans. In Brazil, the Caucasus and the Mediterranean basin, for instance, canine leishmaniasis has long been identified as both a public health risk and a veterinary challenge, yet intersectoral response approaches remain fragmented [35,36,37]. Likewise, *T. cruzi*, the causative agent of Chagas disease, maintains enzootic cycles in wild and peridomestic mammals, including rodents, armadillos, and opossums, with domestic animals serving as the bridging hosts [38]. Even where domestic vector control has been successful, it is only temporary as sylvatic reservoirs and environmental disturbances repeatedly drive reinfestation and resurgence of the disease [23,39]. Additionally, *T. b. gambiense* and particularly *T. b. rhodesiense*, the causative agents of human African trypanosomiasis (HAT), also infect both livestock and wildlife, with tsetse flies enabling transmission between these animals and humans [40,41]. While the contribution of an epidemiologically significant animal reservoir for gambiense HAT remains a matter of debate, computational models developed by Stone and Chitnis (2015) [42] show that the close association between domestic animals and humans contributes to *T. b. gambiense* transmission regardless because the animals act in any case as a ‘sink’ for tsetse bites. This is particularly relevant because tsetse can carry mixed infections of two or three trypanosome species [43]. In contrast, the importance of wild and domestic animal reservoirs for rhodesiense HAT is very well documented [41]. Furthermore, its veterinary counterpart, AAT (known as nagana in Africa), is a major cause of livestock mortality and morbidity in sub-Saharan Africa. This results in great economic losses yet receives almost no attention in pharmaceutical research and development pipelines [26].

These examples demonstrate the limitations of a siloed, anthropocentric strategy to disease control. A One Health view instead advocates an all-inclusive and coordinated intervention that addresses all aspects of the transmission cycle. This includes the coordination of joint human–animal treatment campaigns, development and licensing of veterinary antiparasitics, incorporation of ecological and vector data into planning, and creation of a shared monitoring platform.

Furthermore, anthropogenic drivers, including agricultural encroachment, deforestation, mining, and climate change, are reshaping vector behaviour and distribution. These forces alter the landscape of disease ecologies. An example is the northward spread of leishmaniasis into southern Europe and the emerging cases of Chagas disease in North America associated with migrating vectors [10,44,45,46].

From a perspective of pharmaceutical innovation, embedding the One Health strategy in drug discovery and delivery means restructuring pipeline priorities. This includes investing in dual-use drugs apt for both humans and animals, such as isoxazoles, nucleosides and oxaboroles, as well as modifying drug formulations for veterinary use [47,48,49]. State-of-the-art delivery systems such as long-acting injectables or sustained-release implants could solve the very real problem of the need for repeated administration in underserved rural and nomadic animal populations [50].

Also, the One Health paradigm stresses the need for environmental risk governance in the deployment of pharmaceutical products. This is important because residual antiparasitic compounds may disrupt insect vectors, bioaccumulate in non-target species, or alter soil microbiota [51,52]. Hence, drug development must be One-Health-aligned, involving environmental impact assessments, safe disposal protocols, and ecotoxicological frameworks.

In summary, the transmission ecology and the biology of kinetoplastid parasites require a One Health approach. Beyond theoretical appeal, this approach offers meaningful strategies for pharmaceutical innovations that are scientifically robust, environmentally sustainable, and socially inclusive. It is through this lens that the remainder of this review critically assesses current and emerging pharmacological interventions.

## 3. Overview of Existing and Emerging Pharmaceutical Interventions

In the absence of vaccines, pharmaceutical interventions have long been the mainstay of clinical response to kinetoplastid diseases. Vector control has been particularly important to reduce transmission of *T. cruzi* (see Section 5) and it is more impactful because the available Chagas disease therapies are so inadequate. Indeed, the existing anti-kinetoplastid pharmacological armamentarium, its deployment, and the design of novel therapeutics remain both therapeutically unsatisfactory and structurally fragmented. Most of the anti-trypanosomatid agents currently in use were developed decades ago, with their mode of action incompletely understood, in addition to being prone to resistance [16,18,53]. Almost all of the drugs currently used for human kinetoplastid infections were developed with an anthropocentric focus, neglecting the complex but crucial ecological networks and zoonotic cycles that maintain disease transmission [54,55]. It is in this context that this section provides a comprehensive and comparative overview of approved drugs and investigational compounds for HAT, Chagas disease, and leishmaniasis, at the same time highlighting some of the veterinary blind spots. It is organised around their pharmacodynamic characteristics, chemical properties, and translational significance within a One Health framework.

### 3.1. Leishmaniasis

Leishmaniasis consists of a range of clinical syndromes, including visceral (kala-azar), cutaneous (CL), and mucocutaneous leishmaniasis (MCL). Over 20 *Leishmania* species are known to cause disease in humans. The variety of disease manifestations highlights the complex interplay between the host immune system, parasite virulence, and ecological exposure [56]. In addition to these three primary manifestations, unsuccessful treatment of visceral leishmaniasis (VL) can give rise to a form of leishmaniasis known as post-kala azar dermal leishmaniasis (PKDL) that is very distinct from the principal CL that is limited to site of infection; the absence of an effective cellular immune response can further give rise to diffuse cutaneous leishmaniasis [1]. The many species and the different manifestations even with the same species highly complicate treatment and the chemotherapeutic management of the infection remains disease-form-specific and exceedingly region-dependent. Additionally, treatments are often challenged by adverse effects, drug resistance, and suboptimal field compatibility [57,58].

#### 3.1.1. Current Therapeutics for Leishmaniasis

Antimonials

**Figure 1:**
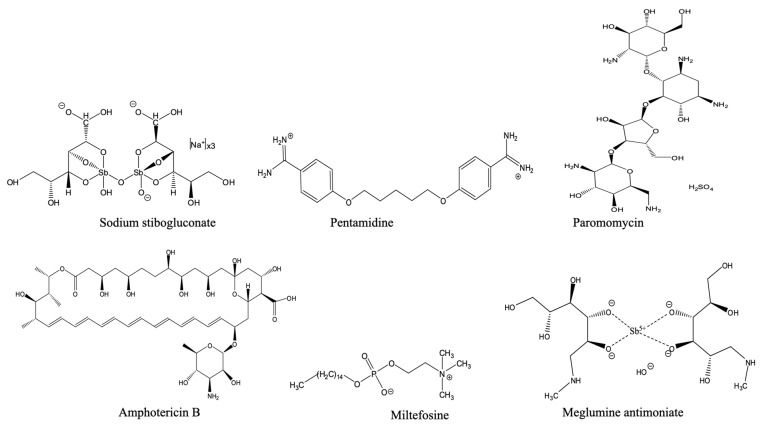
Current therapeutics for human leishmaniasis. Shown are the main drugs in clinical use—pentavalent antimonials, amphotericin B formulations, miltefosine, and paromomycin. They act through diverse mechanisms but are limited by toxicity, resistance, and accessibility challenges, underscoring the need for safer and more effective One-Health-aligned therapies.

The pentavalent antimonials meglumine antimoniate and sodium stibogluconate (Figure 1) have been in use as the cornerstone of antileishmania therapy for over seven decades. However, their cellular and molecular modes of action remain poorly understood. The active form is almost certainly the reduced trivalent form Sb(III) and it is in this valency that the drug is taken up by the aquaporin AQP1, mutations which give rise to antimonial resistance [59,60]. Sb(III) has been shown to then inhibit the parasite’s trypanothione reductase and interrupt energy metabolism [61] but the antileishmanial action of the Sb(V) prodrugs is based on the selective accumulation, first into the infected macrophage’s phagolysosome and then into the parasite as Sb(III), where they appear to exert a polypharmacological toxicity. These drugs have serious drawbacks, including the need for intravenous administration, toxicity issues (such as pancreatitis and cardiotoxicity), and growing resistance, especially in the Indian subcontinent [62,63].

Pentamidine

Pentamidine, an aromatic diamidine (Figure 1), was introduced in the 1940s, as an alternative for the more toxic diamidine stilbamidine for the treatment of visceral leishmaniasis [64]. Pentamidine isethionate and dimesylate, the two formulated salts of pentamidine, were used as a second-line treatment to overcome the increasing issue of antimonial resistance, particularly for cutaneous leishmaniasis caused by *L. guyanensis* and *L. panamensis* in South America [65]. However, their clinical use is limited due to serious toxicity issues, including disturbances in glucose metabolism resulting in hypoglycaemia, nephrotoxicity, hypotension, gastrointestinal and cardiotoxicity, and high treatment failure rates in some regions, especially in India [58,66]. Moreover, the effectiveness of pentamidine depends on the infected Leishmania species and the geographical location. Clinical success rates are inconsistent and can range from as low as 35% for *L. braziliensis* in Peru [67] to 96% in Colombian cases of cutaneous leishmaniasis [68].

Notwithstanding pentamidine’s established use, its mode of action remains to be fully understood. The mechanism of action of pentamidine in *Leishmania* is nonetheless considered to be via disruption of nucleic acid and protein synthesis [58]. Pentamidine is also reported to inhibit the synthesis of polyamines [69], which impacts cell division and antioxidant protection. It further interferes with mitochondrial function by targeting kinetoplast DNA, respiratory chain complex II, and inhibiting mitochondrial topoisomerase II [70,71], and rapidly depolarises the mitochondrial membrane potential [72]. Further evidence for a mitochondrial target comes from observations of pentamidine-treated cells with electron microscopy, showing disruption of the mitochondrion and kinetoplast mere hours after treatment [73].

It has been proposed on the basis of a detailed study of pentamidine uptake in *L. donovani* and *L. amazonensis* that pentamidine is rapidly internalised by the parasite by a plasma membrane transporter, with a K_m_ of 7.4 µM, and subsequently from the cytosol into the mitochondrion [74]. The accumulation into the mitochondrion of dicationic pentamidine is driven by the mitochondrial membrane potential (MMP) and reduced MMP is associated with pentamidine resistance [72,74]. It has become clear that the efficacy of pentamidine against *Leishmania* spp., compared to *T. brucei* spp., is limited predominantly by the modest efficiency of its uptake, as expression of the high-affinity pentamidine transporter of *T. b brucei*, TbAQP2 (K_m_ = 36 nM [75] in *L. mexicana* promastigotes greatly increased in vitro pentamidine uptake and sensitivity [76]. Thus, like the antimonial prodrugs, pentamidine appears to be a general toxin with polypharmacology that derives its selective antiprotozoal activity from preferential uptake into the parasite over the host cells.

Paromomycin

Paromomycin, an aminoglycoside antibiotic (Figure 1), is an antileishmanial agent that was originally isolated in the 1950s as a secondary metabolite from filtrates of *Streptomyces krestomuceticus* (*Streptomyces rimosus*). Its efficacy was first observed in murine models of leishmaniasis in 1961; clinical validation then followed in the 1990s, which led to the development of an affordable intramuscular formulation by the Institute for OneWorld Health [58]. Although no longer used as an antibiotic, in 2007, it was licensed in India as a well-tolerated, effective, and affordable chemotherapy for VL [77].

Paromomycin acts by binding to the 30S ribosomal subunit. Once bound, it disrupts protein synthesis through interaction with the 16S rRNA [78]. Cryo-EM studies confirm that its ribosomal binding interferes with tRNA positioning [79]. It also exerts its antileishmania activity by affecting the mitochondrial membrane potential and respiration [80]. Paromomycin is administered intramuscularly for visceral leishmaniasis or topically for cutaneous leishmaniasis, where it shows up to 95% efficacy, particularly in East Africa and South Asia [81,82].

While generally safe, it may cause localised pain, transient hearing loss, or nephrotoxicity. To improve its therapeutic index, advanced delivery platforms, such as liposomes, microspheres, hydrogels and solid lipid nanoparticles are under development [83].

Amphotericin B

Amphotericin B, like paromomycin, is a 1950s antibiotic isolated from a *Streptomyces* species. It is an amphiphilic polyene agent (Figure 1), that disrupts fungal and leishmanial membranes by binding ergosterol [84] at lower concentrations than the corresponding sterol, cholesterol, in host cells [85]. This interaction effectively leads to ion-conducting pores in the plasma membrane and cell death [86,87]. Toxicity issues exist, but the liposomal amphotericin B (AmB) formulation (AmBisome) is still the best therapeutic choice for visceral and cutaneous leishmaniasis. Nevertheless, the need for cold-chain storage presents a challenge, presenting a logistical challenge for deployment in remote endemic regions [88,89]. In addition, its efficacy depends on the clinical manifestation, the patient’s immunological status, and the endemic region. Besides its side effects, high cost and the need for parenteral administration considerably limit its use in developing countries [90].

Miltefosine

Miltefosine, an alkylphosphocholine (hexadecylphosphocholine, Figure 1), was discovered as a potential anticancer drug in the early 1980s but it was soon discovered to have antileishmanial activity as well [91], and a series of clinical trials between 1996 and 2004 led to its registration as the most recent, and the first oral antileishmanial [92,93]. Importantly, it was developed as the result of a public–private partnership (PPP) involving the Drugs for neglected Diseases initiative (DNDi.org) and the pharmaceutical company Zentaris GmbH [92]. Although the mechanism of its antileishmanial activity is not comprehensively understood, it is understood that its structural similarity to membrane phospholipids makes the cell membrane the main site of action, where it alters membrane lipid metabolism by inserting into the phospholipid membrane via interaction with membrane sterols and through miscibility [94]. There are also reports of changes to cellular lipid metabolism [95,96], disruption of intracellular Ca^2+^ homeostasis [97], modulation of host immune responses [98] and induction of apoptosis-like programmed cell death [99]. However, in their very recent review by Zhang et al. (2025) [58], these authors lay out the case that the main effect of miltefosine in vivo is through interference with the leishmanial activation of a specific host signalling pathway, involving phosphatidylinositol 3-kinase (PI3K) and the PKB/Akt protein kinase, that is essential for the parasite’s survival, leading to apoptosis.

The antileishmanial action of miltefosine is dependent on intracellular uptake after the drug associates with lipid rafts in the outer leaflet of the plasma membrane [100]. The drug is then translocated into the cell by a flippase-like activity mediated by a complex comprising a P-type ATPase known as the miltefosine transporter (MT) and a β-subunit called Ros3 [101]. Mutations in MT have been widely linked to miltefosine resistance [101,102].

The use of miltefosine for antileishmanial chemotherapy is considered valuable due to its ease of administration and its reduced toxicity relative to other antileishmania agents like pentavalent antimonies [103]. However, GI toxicity, teratogenicity, and the long half-life of the drug pose a challenge. In addition, resistance is already emerging globally [104,105,106]. It has also been reported to cause toxicity in mammalian cells via the oxidation of DNA bases, apoptosis, and necrosis [107]. Effective oral medication against leishmaniasis is crucial in the fight against the disease, as it is predominantly spread in poor regions and oral administration would enhance adherence to the treatment. Miltefosine (as well as Sb(V) and amphotericin B) is sometimes used off-label in canine leishmaniosis [108] and the effective treatment of canine leishmaniosis is certainly important with in any One Health control strategy. On the other hand, given the apparent ease with which miltefosine resistance emerges, relapse after treatment in dogs raise the question of its long-term efficacy and the risk of human reinfection with resistant strains. Currently, canine leishmaniosis is predominantly treated with allopurinol, a drug that is rarely used for human forms of leishmaniasis, although allopurinol is a leishmaniostatic agent, i.e., it suppresses the parasite’s proliferation but does not achieve parasitological cure [109]. This has potential implications for disease transmission and control.

The veterinary dilemma

Despite the proven utility of the above-described antileishmanial drugs in human medicine, there is scant research into their application for canine leishmaniasis and, perhaps more importantly, the development of new, effective veterinary antileishmanials. One long-term clinical study compared treatment regimens of allopurinol with either miltefosine or meglumine antimoniate and concluded after 6 years of follow-up that the latter produced superior outcomes [110].

The development of effective, curative antileishmanial agents for veterinary use is essential given that dogs are the primary reservoirs for zoonotic visceral leishmaniasis, especially in the Middle East, Latin America, and the Mediterranean. The absence of targeted veterinary formulations and dosing protocols constitutes a significant oversight and addressing this gap could significantly improve disease control at the human–animal interface and strengthen One-Health-aligned treatment strategies. The dilemma is whether to advocate for effective treatments of human and animal leishmaniases with a single drug (combination) or to aim for separate treatments for humans and animals. Given the scale and prevalence of leishmania in not just canines but also felines and rodents and the risk of treatment insufficiency leading to drug resistance, the more prudent option would be to prioritise separate drugs.

#### 3.1.2. Emerging Antileishmanial Therapies and Innovations Beyond Traditional Chemotherapy

Advances in antileishmanial therapeutic research aim to overcome the established challenges of drug toxicity, resistance, and accessibility of treatment. A promising repositioned candidate, fexinidazole, originally approved for HAT (see Section 3.3.2), was reported to be effective against *Leishmania*. In murine models of VL, the fexinidazole metabolites (sulfone and sulfoxide, which rapidly form in the mammalian host) achieved >98% suppression of *L. donovani*, similar to that of miltefosine or Pentostam, with favourable oral pharmacodynamics, supporting its potential for development as once-daily oral therapy for VL [111]. Based on this outcome, a Phase II clinical trial (NCT01980199) was conducted in Sudan in 2013 to evaluate fexinidazole for VL. However, the development of fexinidazole as an antileishmania drug was interrupted in 2014 even though all the patients showed clinical improvement (microscopy-based parasite clearance) during treatment. The interruption was due to inconclusive efficacy, as there was relapse in some of the patients after a 6-month follow-up check. Nonetheless, fexinidazole–miltefosine combination therapy is now under investigation, with pharmacokinetic and safety assessments being carried out in healthy volunteers to advance the development of the therapy as a potential oral-only treatment for VL [112]. Another way forward was a proposed reformulation of oral fexinidazole with a self-emulsifying drug delivery system (SEDDS) that improved antileishmanial efficacy in mice [113].

**Figure 2:**
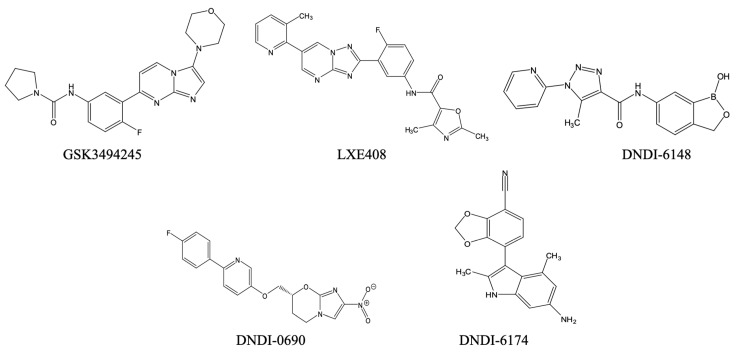
Emerging antileishmanial chemotherapies. The figure highlights some of the promising drug candidates. These agents target parasite-specific pathways and represent advances toward safer, orally available, and field-adaptable therapies.

Parallel efforts are centred on proteasome inhibitors like GSK3494245 and LXE408 (Figure 2), whose modes of action involve the disruption of chymotrypsin-like activity of the *Leishmania* proteasome. These molecules were active in in vivo models of leishmaniasis and showed promising pharmacokinetics, positioning them for Phase I/II testing [114,115]. They are being developed by DNDi for the treatment of VL, in partnership with Glaxo Smith Kline and Novartis, respectively. Their development prioritises field readiness such as oral administration, stability in hot climates and the potential for dispersible paediatric presentations [116]. At this point, to the best of our knowledge, no veterinary applications are considered despite the importance of the canine reservoir for several *Leishmania* species, including *L. infantum*, one of the leading VL species.

DNDI-6148 is a benzoxaborole selected for preclinical evaluation against VL based on >98% parasite reduction and an attractive physicochemical/pharmacokinetics profile in rodent models [117]. DNDi has also signalled cross-disease potential for *T. cruzi* (see below) [117,118]. Formulation work for DNDI-6148 emphasises oral bioavailability, high-temperature stability, and scalable synthesis [117], which are crucial for local manufacturing in endemic regions.

The nitroimidazole DNDI-0690 (and second-generation analogues) also demonstrates potent activity against VL and CL, with dermal site pharmacokinetics/pharmacodynamics being explored to enable topical and oral options [119]. Notably, analytical methods now quantify the drug at the target skin site, guiding formulation choices (e.g., topical gels/creams, film-forming solutions) for CL where clinic-based parenteral therapy is impractical [119,120]. Such topical strategies have obvious veterinary translatability for canine CL management, with potential pairing to sandfly repellents to reduce disease transmission.

Another candidate in preclinical development by DNDi for VL is DNDI-6174 (Figure 2), a pyrrolopyrimidine inhibitor binding to the ubiquinone reduction (Q_i_) site of the *Leishmania* cytochrome bc1 complex of the mitochondrial electron transport chain [121]. A unique feature of this drug target is that it is encoded by the kinetoplast maxicircles and thus exists in up to 50 copies in the cell and this ought to make development of resistance through mutations in the target enzyme very slow to arise. The bc1 inhibitors are amenable to solid oral forms and displayed high oral availability in animal studies [121]. Across these DNDi programs, the most impactful levers are as follows: (i) simple oral dosing (once-daily or short-course), (ii) heat-stable tablets and paediatric dispersibles to obviate cold chain, and (iii) topical CL formulations with validated dermal pharmacokinetics/pharmacodynamics.

**Figure 3:**
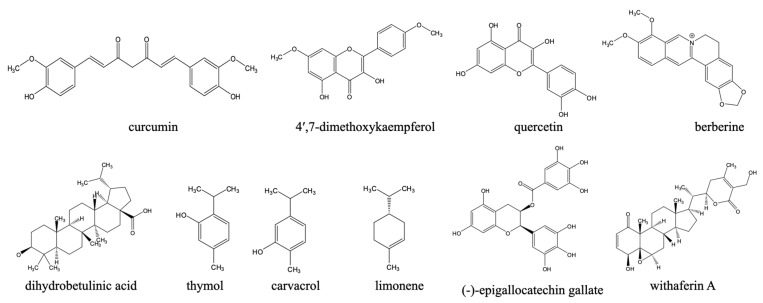
Natural products under investigation for their antileishmanial activities. These natural products provide chemically diverse scaffolds with demonstrated activity against *Leishmania*. Increasingly, natural products are being investigated as adjuncts or alternatives to legacy therapies, offering opportunities for safer, more accessible, and potentially resistance-mitigating options in leishmaniasis management.

Beyond synthetic compounds, natural products (Figure 3) continue to provide chemically diverse scaffolds with activity against *Leishmania* and are increasingly investigated as adjuncts or alternatives to legacy therapies. Their relevance to a One Health agenda is twofold: many are locally accessible within endemic regions (facilitating community uptake and veterinary translation), and several display activities across parasite stages and species that circulate among humans, domestic animals, wildlife, and vectors. Although a comprehensive treatise on this subject is beyond the scope of this paper, the discovery of natural compounds and the promotion of green chemistry approaches are an important part of a sustainable drug discovery approach

For instance, curcumin displayed significant activity against *Leishmania* species. It induced apoptotic-like death in *L. major* promastigotes, and reduced amastigote burdens in macrophages [122]. However, the potency of curcumin and its derivatives against *L. major* promastigotes and *L. mexicana* amastigotes was lower than that against other kinetoplastids like *T. brucei* or *T. evansi* bloodstream forms [123].

Also, kaempferol and its O-methylated derivatives displayed activity against *L. mexicana*. Propolis-derived 4′,7-dimethoxykaempferol (Figure 3) achieved low-micromolar EC_50_ values, motivating exploration of bee-product phytocomplexes as affordable adjuncts in the treatment of human cutaneous form of leishmaniasis [124]. A very complete overview of the effects of flavonoids including kaempferol derivatives against leishmaniasis and Chagas disease was published by Boniface and Ferreira [125].

Another natural compound of considerable interest is quercetin (Figure 3). Quercetin, its derivatives, and quercetin-loaded nanoemulsion demonstrate leishmanicidal effects in experimental visceral Leishmaniasis via interference with iron metabolism and ribonucleotide reductase. The encapsulation into nanoemulsions further improved anti-*L. donovani* efficacy and biocompatibility [126,127,128].

Among alkaloids, berberine (Figure 3) reduced *L. donovani* burdens in hamsters with lower toxicity than pentamidine, and liposomal berberine has been shown to enhance intracellular delivery. Berberine formulations may be economical and suitable for regional manufacturing [129,130].

In addition, triterpenes such as dihydrobetulinic acid triggered selective apoptosis in *L. donovani* promastigotes and amastigotes by targeting topoisomerases [131] and inspired a significant medicinal chemistry campaign to identify betulin-based analogues with improved potency [132].

Notably, phenolic monoterpenes and phenolics directly relevant to canine reservoirs are gaining traction. The isomers thymol and carvacrol, which can be isolated from multiple medicinal plants and herbs, have shown in vitro activity against *L. infantum*—he principal agent of zoonotic VL—with impressive in vivo safety data [133]. The related compound limonene (Figure 3) demonstrates broad-spectrum antileishmanial activity against *L. amazonensis*, *L. major*, and *L. braziliensis*, with efficacy observed in both in vitro and in vivo models. Treatment with limonene reduces macrophage infection rates and leads to significant decreases in lesion size and parasite burden in murine models. Its proposed mechanisms of action include disruption of plasma membrane integrity through increased membrane fluidity, induction of parasite cell lysis, and potential inhibition of isoprenoid biosynthesis, underscoring its promise as a natural compound with therapeutic potential in leishmaniasis drug development [134].

Similarly, green-tea catechin, (-)-epigallocatechin gallate (Figure 3), was shown to inhibit *L. (L.) amazonensis* and *L. (V.) braziliensis* proliferation, targets the trypanothione pathway, and demonstrates efficacy in mice—an attractive mechanism shared across trypanosomatids [135].

Also, withanolides (e.g., withaferin A) reduced amastigote loads and modulated host cytokines in *L. donovani* models, suggesting dual antiparasitic and immunoregulatory benefits that could shorten therapy or limit relapse [136].

Beyond the recent contributions of synthetic and natural compounds to the antileishmania preclinical developments, other therapeutic innovations show considerable promise. These include targeted heat-based therapies for CL. For example, in Iran, CO_2_ laser treatment showed a superior cure rate (~90–94%) when compared with intralesional antimonials (95% vs. 4.5% side-effect rate), with a much faster lesion resolution and minimal scarring. A similar result was observed in Sudan, where most of the patients (8 out of 10) had either a very good improvement or a complete cure by the end of the follow-up [137,138].

Similarly, thermotherapy using the HECT-CL device (a sodium acetate heat pack) was shown to be safe and efficacious in field settings, with cure rates reported to be around 60% in Peru and other regions of South Asia [139,140].

Moreover, nanotechnology-enabled formulations are increasingly being explored to improve the delivery of established therapies. Solid lipid nanoparticles (SLNs), liposomal forms, polymeric microspheres, and nanotube conjugates are being investigated to deliver paromomycin, amphotericin B, and other antileishmanial agents more selectively to infected macrophages [113,141]. These strategies significantly reduce systemic toxicity, improve bioavailability, and enable lower dosing frequencies. These also enhance the practicability of treatments in resource-poor settings or veterinary contexts [142]. While there is great promise in this strategy, as liposomal formulations of amphotericin B, for instance, have demonstrated, full clinical trials and implementation for most of these experimental ideas remain some way off.

Another critical advance is emerging combination regimens. In India and Sudan, for instance, single-dose liposomal amphotericin B was combined with short courses of paromomycin or miltefosine. This achieved cure rates of over 96–97% in VL patients, including HIV-coinfected patients; at the same time, it reduced hospitalisation and adverse events when compared with monotherapy [143]. Likewise, a study by DNDi and collaborators in Eastern Africa using injectable paromomycin and oral miltefosine showed that the combined therapy was more potent, safe, and had a shorter treatment period for VL, and a reduced number of painful injections—offering significant benefits for patients in endemic regions [144]. The combination of pentavalent antimonials plus paromomycin was also very effective in East Africa, with 93.9% efficacy at 6 months, and is currently the first-line treatment for VL without HIV coinfection in East Africa [123,124,125]. In India, a multitude of studies on dose regimens and drug combinations against VL were prompted by the high levels of pentavalent antimony resistance in the region, centred on Bihar, quickly followed by the declining efficacy of miltefosine monotherapy [145]. However, a DNDi study showed that a single-dose liposomal amphotericin B (10 mg/kb by infusion) was >90% effective [146] and this remains the standard treatment for now [147].

These developing therapeutic approaches, ranging from repurposed oral drugs and proteasome inhibitors to heat-based interventions, nanodelivery systems, and combination treatments, reflect a multi-pronged innovation ecosystem. In combination with the One Health framework, these strategies hold promise for safer, shorter, and more scalable treatment options that is aligned with global disease elimination goals.

### 3.2. Chagas Disease

Chagas disease (American trypanosomiasis) is a vector-borne disease caused by the kinetoplastid parasite *T. cruzi*. It affects an estimated 6–7 million people globally, predominantly in Latin America. The infection is chronic and may or may not lead to severe clinical disease over a period often measured in decades [148]. There is now an increasing concern due to the incidence of the disease in non-endemic regions mainly due to climate change expanding vector habitat and through migration of infected people [15,149]. The disease is transmitted mainly through the faeces of triatomine bugs (commonly known as kissing bugs). *T. cruzi* can also spread via blood transfusion, organ transplantation, congenitally, and orally through contaminated food and drink [150,151,152,153].

From a One Health perspective, *T. cruzi* exemplifies a pathogen deeply embedded in a multi-host ecology. There are over 150 mammalian reservoir species, which range from wild marsupials and rodents to domestic dogs, livestock and cats [154]. For many of these species, pathological progression is too slow to lead to severe symptoms, but myocarditis has been reported in dogs [155] and neural symptoms in equines [156]. As animals are thus largely symptomatic carriers of this zoonotic parasite, no veterinary therapeutics or standardised treatment protocols have been developed, and the vast wild and domestic animal reservoir is largely ignored. However, as pointed out by Desquesnes et al. (2022) [157], accidental transmission can happen when handling animal carcasses or samples in much of the Americas reaching from South America to halfway into the USA.

#### 3.2.1. Current Therapies for Chagas Disease

**Figure 4:**
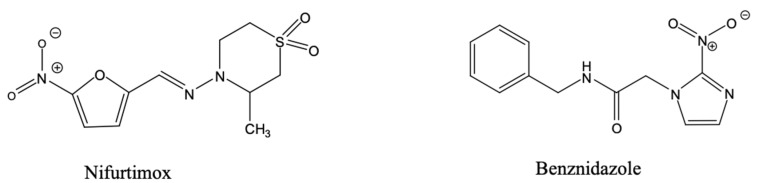
Current chemotherapies for Chagas disease. Present treatments remain limited to the nitroheterocyclic compounds benznidazole and nifurtimox, which act through nitro-reductive activation generating toxic intermediates. While effective in acute infections, both are hampered by toxicity, poor tolerability, and reduced efficacy in chronic disease, underscoring the need for improved options.

The treatment for Chagas disease is currently restricted to two nitroheterocyclic drugs, benznidazole and nifurtimox (Figure 4). These two drugs were developed well over half a century ago.

Benznidazole is a nitroimidazole and it is the first-line agent, particularly in acute infections and paediatric patients. Its mechanism of action involves metabolic activation by parasite-specific nitroreductases [158], which generates reactive metabolites, free radicals such as superoxide, that then react with and disable DNA, lipids and proteins in the parasite [159]. Although it derives its selectivity from the activation by the trypanosome-specific nitroreductases, a lower, spontaneous rate of radical production in host tissues contributes to a high incidence of adverse reactions, including gastrointestinal disturbances, peripheral neuropathy, dermatitis, and leukopenia [160].

Nifurtimox, though a nitrofuran derivative, is also a substrate of the trypanosomal nitroreductases [158,161] and similarly induces oxidative stress within the parasite. However, it is less preferred due to the need for longer treatment courses and lower tolerability. Both drugs are most effective during the acute phase of the disease and show reduced potency in chronic infections, where parasite loads are low and immunopathological processes dominate [162]. A major issue is that nifurtimox and benznidazole, the only two registered drugs against Chagas disease, have the same mechanism of action and are, as a result, cross-resistant [158,163]—meaning that resistance to either drug loses every treatment option to a fatal disease.

There has been much reporting that the large genetic variation within *T. cruzi*, giving rise to a classification into six discrete typing units with different geographical distributions (DTUs, TcI–TcVI [164], means that there is much variation in sensitivity to the nitroheterocyclic drugs. A recent meta-analysis of 60 articles certainly confirmed a variable benznidazole sensitivity across the different genotypes and highlighted lower sensitivity in TcI than TcII but also noted that the analysis was complicated by the lack of assay standardisation between studies [165]. A further limitation of the reliance on these old drugs is that long treatment regimens give rise to many adverse drug reactions (ADR)s, with patients having an 80% chance of one or more ADR during nifurtimox treatment [166], leading to interruption of treatment in up to 75% of patients [167].

Treatment failure rates have been reported, particularly in adults with chronic disease, and long-term parasitological cure is rarely achieved [168]. Although the drugs reduce parasite burdens and antibody titres, their effects on reversing, or even slowing the progress of, the cardiomyopathy of Chagas disease and even on mortality remain in question [169]. Despite all these limitations, benznidazole and nifurtimox remain the only approved therapies to date (and no drugs have been approved for veterinary treatment of *T. cruzi* infection. The perception of the imbalance of adverse effect versus benefit of treatment with benznidazole or nifurtimox is such that it was estimated in 2009 that fewer than 1% of people infected with *T. cruzi* have received etiological treatment, making treatment for Chagas disease one of symptom management rather than attempted cure [170]. This situation has still not materially changed.

#### 3.2.2. Pipeline for Anti-Chagas Disease Candidates and Translational Challenges

Recent drug development efforts aimed to develop safer and more effective alternatives to the nitroheterocyclic drugs. One strategy to shorten the drug discovery timeline is through drug repurposing. Fexinidazole, which was originally developed for HAT, demonstrated curative activity against *T. cruzi* in acute and chronic *T. cruzi* murine models [171]. However, the initial results in a multicentre randomised double-blind, Phase 2 trial were unsatisfactory as the treatment did not meet the primary endpoint of sustained negative PCR for *T. cruzi*, although parasite loads had initially decreased sharply upon treatment [172]. This has stopped clinical development of fexinidazole for Chagas disease, at least as monotherapy and for the time being.

**Figure 5:**
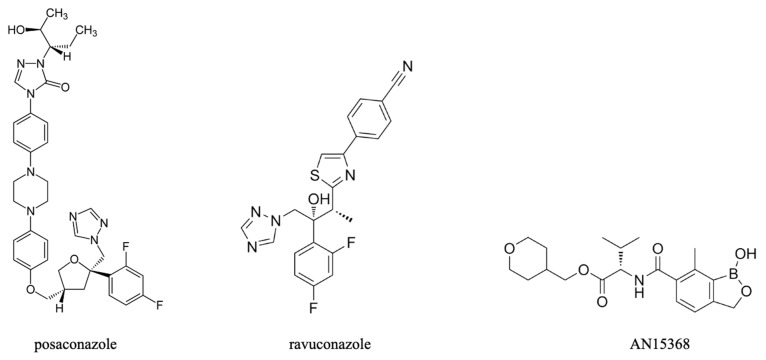
Pipeline for anti-Chagas drug development. The figure includes two compounds in development, triazole antifungals, posaconazole and ravuconazole, which inhibit ergosterol biosynthesis—an essential pathway in *T. cruzi* since the parasite cannot utilise mammalian cholesterol—and AN15368, which targets a kinetoplast endonuclease called cleavage and polyadenylation specificity factor 3 (CPSF3). These, alongside LXE408, and DNDi-6148, exemplify efforts to expand safer, more effective therapeutic options for Chagas disease.

Posaconazole and ravuconazole (Figure 5) are triazole antifungals that inhibit ergosterol biosynthesis, which is an essential pathway in *T. cruzi* as they are unable to use mammalian cholesterol [173]. Early in vitro and in vivo tests against *T. cruzi* were encouraging but also highlighted significant differences in responsiveness among parasite strains [174]. Clinical trials with posaconazole and ravuconazole, however, were disappointing, with inferior performance relative to benznidazole [175] and there was no demonstrable benefit from a combination of posaconazole and benznidazole over benznidazole monotherapy [176]. Thus, the effort to repurpose the azole class of antifungals appears to have also been unsuccessful.

Novel compounds, with unrelated mechanisms of action, however, may emerge for the screening of chemical libraries. Among these are series of promising inhibitors of the chymotrypsin-like activity in kinetoplastid proteasomes. The most advanced compound in this series is LXE408 (Figure 2), which displays an IC_50_ for the *L. donovani* proteasome and EC_50_ against intracellular amastigotes of just 40 nM [115]. According to unpublished results related by [177], LXE408 displays potent activity against intracellular *T. cruzi* amastigotes and has performed well in preclinical and Phase 1 clinical trials, leading to preparations for a Phase 2 trial.

Two further leads are from the benzoxaborole class, AN15368 (Figure 5), and DNDI-6148 (Figure 2). Both target a kinetoplast endonuclease called cleavage and polyadenylation specificity factor 3 (CPSF3) [117,178]. AN15368 was shown to effect complete sterile cure in rhesus macaques naturally infected with *T. cruzi* of diverse genetic types without overt toxicity after 60 days of treatment [179]. DNDI-6148, which is being advanced for the treatment of visceral leishmaniasis, is also being considered a promising candidate for the treatment of Chagas’ disease [177,180]. DNDI-6148 was shown to be well tolerated and safe after a single oral dose in the cohort of the first-in-human study that was completed in 2022. However, further clinical trials of DNDI-6148 are currently on hold pending further studies to ascertain the potential for reproductive toxicity [181].

DNDi’s “UW series”, originating from the University of Washington and being further developed in partnership with the university of Dundee and GSK, produced single-compound cures in chronic Chagas bioluminescent mouse models without relapse after immunosuppression, situating the series among the most advanced discovery-stage assets for chronic disease [182]. Separately, bc1 Q_i_-site inhibitors from a pyrrolopyrimidine series, in combination with benznidazole, delivered 5-day short-course cures in chronic models [183], spotlighting the feasibility of abbreviated oral regimens more compatible with primary care delivery. These approaches provide foundations for fixed-dose combinations (FDCs) to minimise resistance and further shorten therapy.

Though these compounds show real promise against *T. cruzi* and animal models of Chagas disease, none have been tested for veterinary application for *T. cruzi* infection, and we are not aware of any efforts to develop such treatments.

**Figure 6:**
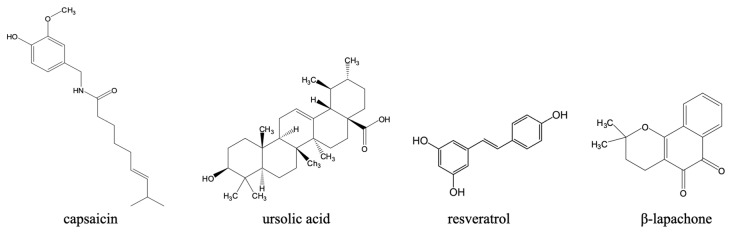
Anti-Chagas disease natural compounds under investigation. Shown are emerging agents under investigation for activity against *T. cruzi*. These candidates target parasite-specific pathways and aim to overcome the limitations of benznidazole and nifurtimox, offering prospects for safer, more effective, and potentially curative therapies for both acute and chronic Chagas disease.

Beyond synthetic compounds, there are also important contributions from natural compounds (Figure 6) to the preclinical development of anti-Chagas drugs. For example, capsaicin, a vanilloid alkaloid from *Capsicum* spp., showed nanomolar trypanocidal activity against *T. cruzi*, with trypomastigotes more susceptible than epimastigotes [184]. Its mechanisms were reported to involve mitochondrial dysfunction, arginine kinase inhibition, and interaction with prohibitin-2, implicating apoptosis induction [184,185]. Remarkably, capsaicin was 57-fold more potent than benznidazole, the current Chagas disease therapy [185]. Pharmacokinetic studies also confirm that orally administered free or liposomal capsaicin achieved plasma concentrations exceeding the IC_50_ for *T. cruzi*, supporting its suitability as an oral treatment [186]. Combined with low cost, stability, and established safety, capsaicin represents a compelling candidate for novel, accessible therapies against Chagas disease within endemic regions.

Also, several polyphenols and terpenoids have shown promise against *T. cruzi.* One such example is ursolic acid, a pentacyclic triterpene from edible plants, which reduced intracellular amastigote loads in macrophages and cardiomyocytes by promoting host autophagy and exerted a direct trypanocidal effect on trypomastigotes; efficacy was also confirmed in murine models [187]. Given orally, ursolic acid displayed good in vivo activity against the Y-strain of *T. cruzi*, with a 50 mg/kg dose reducing peak parasitaemia by 79% [188].

Another example is resveratrol, which was reported to inhibit *T. cruzi* arginine kinase and improve cardiac conduction abnormalities in chronically infected mice, positioning it as a host-directed adjunct with antiparasitic activity [189,190].

Naphthoquinones remain among the most extensively studied natural scaffolds and β-lapachone derivatives displayed strong trypanocidal activity across life stages. Formulation strategies (e.g., 2-hydroxypropyl-β-cyclodextrin encapsulation) addressed the solubility and toxicity constraints observed with the parent compound, β-lapachone [191]. Epimastigote death upon treatment with the β-lapachone derivative R72 involved selective apoptosis, autophagy, and necrosis [192].

These *T. cruzi* leads readily align with a One Health framework. It may be possible to evaluate host-directed phytochemicals like ursolic acid and resveratrol, sourced from food or medicinal plants, in canine Chagas foci (where present) to reduce household reservoirs, and the cultivation and processing of the medicinal plants can be carried out in endemic regions to support sustainable supplies.

#### 3.2.3. Perspectives on the One Health Limitations of Current Chagas Disease Therapies

The failure to develop veterinary treatments against animal infections with *T. cruzi* shows the inherent structural human bias in Chagas disease chemotherapy. This is understandable as acceptable and effective treatments for Chagas disease are still lacking and urgently needed, and because the slow progress of *T. cruzi* infection can mean that infected animals do not go on to develop severe clinical disease from the infection. However, canine Chagas disease is associated with cardiac arrhythmia and chronic myocarditis, and sometimes neural pathologies, particularly in younger dogs [193,194].

*T. cruzi* has a wide host range, including over 200 domestic and wild mammalian species [195]. Dogs and other animals that live in proximity to humans and triatomine vectors, serve as amplifying hosts, perpetuate vector infection cycles and increase transmission to humans [196,197]. In rural areas, livestock such as goats, cats, and pigs can also harbour parasites, acting as reservoirs or vector attractants [198]. This is widely recognised, yet no commercially available tests to detect *T. cruzi* in domestic animals exist [199] and diagnosis is often difficult, especially when parasitaemia is low [193].

Although there are no registered drugs for treating Chagas disease in domestic or wildlife reservoir species, benznidazole has been used off-label to treat canine CD, with nifurtimox being too toxic, but even this drug leaves serum antibody titres high and, crucially, it is unclear whether treatment, especially in the symptomatic, chronic phase, has any clinical benefit for the infected animal—even if it should be parasitologically effective [193]. As in human patients, treatment in advanced Chagas disease is necessarily directed towards managing symptoms, especially the cardiac ones. In the final analysis, this is why no veterinary drugs against CD are being developed: the animals, if they present with clinical disease at all, do so in the advanced stages at which treatment of the pathogen will no longer affect the outcome. As such, it is doubtful whether animal owners would pay for such treatment and hence there is no market for such a drug.

The absence of veterinary treatments leaves an important ecological niche unaddressed. This omission is especially an issue in ecotones where human activity encroaches upon sylvatic habitats [200], such as in deforested regions of the Amazon basin. This potentially facilitates spillover into human communities. Logically, if animal CD cannot be treated by conventional antiprotozoal therapies, it must be prevented by diminishing contact between animal and the triatomine vectors. The traditional spraying with insecticides, however, is ecologically unsustainable except at a small domestic scale. An emerging alternative is the application of systemic insecticides such as fluralaner to animals, which very effectively kills triatomines, including pyrethroid-resistant ones, feeding on the animal [201]. This strategy effectively reduces triatomine infestations and thereby the domestic transmission cycle [202] and is a very good example how a One Health approach can safeguard human, animal and environmental health at the same time.

### 3.3. Human African Trypanosomiasis (HAT)

Human African trypanosomiasis (HAT), also known as sleeping sickness, is a devastating disease that occurs in two forms: the chronic and acute forms. It is classified by the WHO as a neglected vector-borne parasitic disease. The disease is caused by two subspecies of *T. brucei*. *T. b. gambiense*, found in West and Central Africa, and it is responsible for up to 98% of all reported cases, while *T. b. rhodesiense*, which is predominant in East and Southern Africa, accounts for about 2%. Both subspecies are transmitted by tsetse flies (*Glossina* spp). HAT mainly affects impoverished rural populations with reduced access to diagnosis and treatment. Although global cases have declined considerably recently due to sustained surveillance and treatment programmes, sporadic outbreaks and underdiagnosis remain a threat, especially in unstable regions [203].

From a One Health standpoint, HAT highlights the interaction between human, animal, and environmental health. The disease is entrenched within a complex transmission ecology involving livestock, wild animals, and vector species that are sensitive to land-use and climatic changes. Zoonotic reservoirs and insufficient veterinary drug development are a barrier to disease elimination [26], especially for rhodesiense HAT but likely also for the gambiense variant, as infection risks correlate with the density of pigs and/or cattle [204].

#### 3.3.1. Historical Therapeutics: Arsenicals and Eflornithine

**Figure 7:**
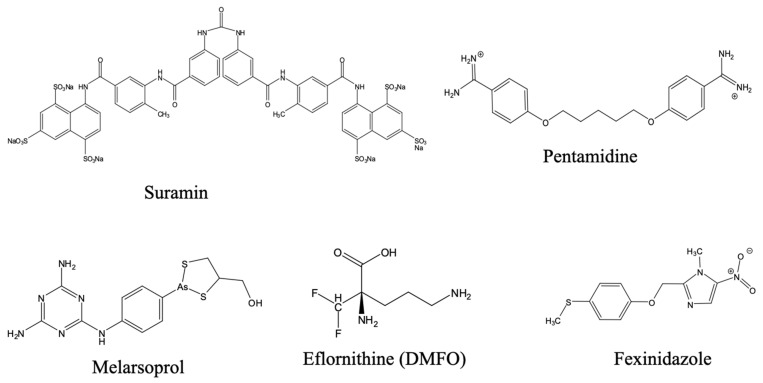
Current drugs for treating human African trypanosomiasis (HAT). Shown are approved therapies for (HAT). These remain central to disease control despite toxicity, resistance, and access challenges.

For much of the 20th century, chemotherapeutic options were highly toxic and limited [16]. An arsenic derivative, melarsoprol (Figure 7), developed from earlier arsenical drugs like tryparsamide and introduced in 1949, was used to treat neurological-stage HAT. Its use was immediately marred by severe toxicity, including reactive encephalopathy, with a high mortality rate among those treated [205]. Despite its efficacy, melarsoprol’s adverse toxicity profile, together with the need for intravenous administration, made it challenging for routine deployment in resource-poor and remote settings and increasing resistance further highlighted the need for alternative treatments [206]. Pentamidine has been used for first stage, haemolymphatic gambiense HAT since its introduction in the late 1930 [207], and suramin for early stage rhodesiense HAT [208].

The introduction of eflornithine (α-difluoromethylornithine) in the 1990s for the treatment of the second stage of HAT caused by *T. b. gambiense* marked a therapeutic breakthrough. Eflornithine, an amino acid analog, covalently inhibits ornithine decarboxylase, thereby disrupting polyamine biosynthesis, which is needed for parasite survival [209]. It was an improvement over melarsoprol but required a burdensome 14-day intravenous regimen and still had severe, if not fatal, side-effects. A much shorter and less toxic schedule was introduced with the nifurtimox–eflornithine combination therapy (NECT). This strategy drastically reduced the infusion frequency and halved the hospital stay. Nevertheless, NECT still requires inpatient care, trained personnel, and sterile supplies. This still poses a substantial challenge for implementation in endemic, resource-poor areas [210].

The recent development and registration of fexinidazole, which is the first all-oral therapy for HAT, signifies a paradigm shift in the fight against HAT. Fexinidazole, like benznidazole and nifurtimox, is a 5-nitroimidazole prodrug, which is metabolised by trypanosomal nitroreductases to the active compounds that are believed to cause DNA and protein damage in parasites through free radicals [211]. It is effective against both the early (haemo-lymphatic) and late (meningo-encephalitic) stages of the HAT caused by *T. b. gambiense* and *T. b. rhodesiense*. Its effectiveness against both stages of rhodesiense HAT means that there is no longer a requirement for a lumbar puncture for staging the disease and the oral formulation means hospitalisation is no longer required either, important in resource-limited communities. Fexinidazole has now been approved by the European Medicines Agency, the WHO has approved it for both gambiense and rhodesiense HAT, and several endemic countries have also approved it for use. However, it is only recommended for use in patients aged 6 years and above with a bodyweight of 20 kg or more [212] and pharmacokinetics in immunocompromised or coinfected patients require further evaluation.

#### 3.3.2. Recent Advances: Acoziborole (SCYX-7158)

**Figure 8:**
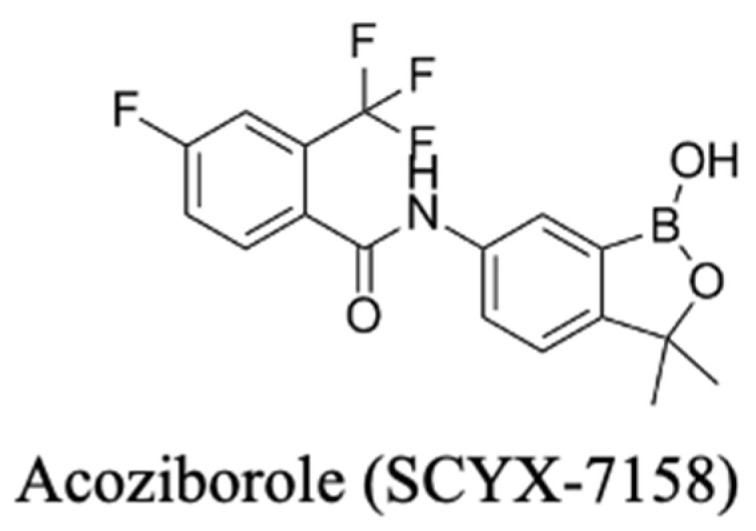
Chemical structure of acoziborole (SCYX-7158). Acoziborole is a benzoxaborole compound that represents a major advance in HAT therapy. Its chemical structure underpins CPSF3 inhibition, disrupting mRNA processing, and it is effective as a single oral dose against *T. brucei gambiense*. Its long half-life, oral availability, and simplified regimen highlight its potential to transform treatment access and field applicability.

Acoziborole (Figure 8), a member of the benzoxaborole compound class, is in the last stages of drug development. Its Phase II/III clinical trials results are promising, where it showed a high degree of efficacy with a favourable safety profile as a single-dose oral treatment for both stages of gambiense HAT. It is reported to specifically target trypanosomes CPSF3, a subunit involved in mRNA processing [48]. It is anticipated to become an available drug within a few years [213,214]. Its long half-life and ability to penetrate the CNS make it ideal for both early- and late-stage HAT; thus, like fexinidazole, acoziborole does not require lumbar punctures for staging, simplifying diagnosis, drug deployment, and treatment. The drug originated from DNDi partnerships and was recently reported to be 95% effective against *T. b. gambiense* as a single oral dose of 960 mg, along with an admirable safety profile [48]. Its development path exemplifies DNDi’s strategy to optimise for oral exposure and long half-life and design a regimen compatible with rural care pathways (no staging lumbar puncture, minimal monitoring) [48]. This template is highly relevant to agents still in preclinical stages that aim for heat-stable, once-only, or short-course dosing suitable for primary care delivery and community campaigns.

With acoziborole, at the time of writing this report, in the final stages before approval, and the recent approval and deployment of fexinidazole, sleeping sickness treatment has made a giant leap forward in less than a decade, after well over a century of inadequate and toxic drugs [16,215], especially for the late stage of the disease, and, as a result, elimination of transmission of gambiense HAT transmission may be achievable. However, challenges remain, as do the tsetse flies, as long as there is a parasite reservoir, either in asymptomatic, undetected human carriers or in animals. In line with the One Health principles, none are safe until all are safe.

#### 3.3.3. The Veterinary Blind Spot: African Animal Trypanosomiasis (AAT)

African animal trypanosomiasis (AAT), or nagana, is mainly caused by *T. vivax*, *T. congolense*, and *T. b. brucei*, with further contributions from *T. evansi*, *T. equiperdum* and *T. simiae* [5]. According to the Food and Agriculture Organisation (FAO), nagana affects an estimated 50 million head of cattle across 36 countries. It is responsible for about 3 million deaths in cattle, along with circa 35 million doses of trypanocidal drugs administered yearly. This results in about USD 4.75 billion in annual economic losses, including from reduced productivity, fertility, and draft power [216]. For AAT, livestock serve not only as victims but also as reservoirs of trypanosomes. This facilitates human exposure via tsetse fly transmission, and unlike gambiense HAT, it is not under control and on a trajectory towards elimination, and there has been no introduction of new treatments for decades.

**Figure 9:**
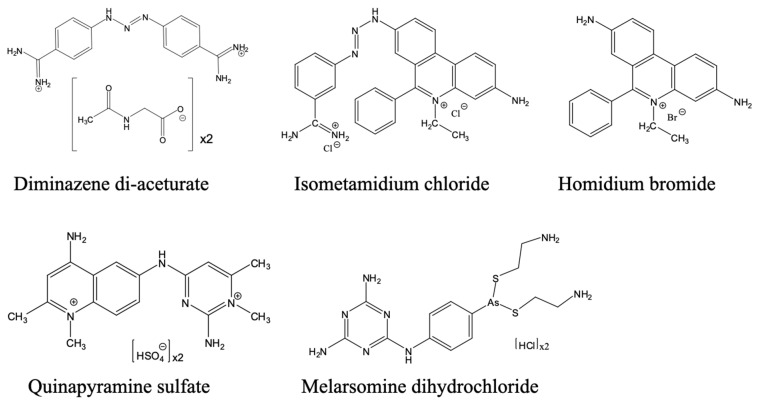
The primary veterinary drugs for AAT. The figure presents the principal trypanocides used against animal African trypanosomiasis (AAT).

AAT remains largely underfunded and under-addressed, as less attention is paid to it. The primary veterinary drugs for AAT are diminazene aceturate, isometamidium chloride, and homidium salts (Figure 9), were developed decades ago and are linked with toxicity, variable efficacy due to counterfeiting, and growing resistance [26]. In addition, these compounds have narrow safety margins, require repeated injections, and lack regulatory harmonisation across the African countries where AAT is endemic. These are in addition to their mechanisms of action and resistance, which are not fully characterised. Outside of Africa, for non-tsetse-transmitted trypanosomiasis, quinapyramine and cymelarsan are also used for some forms of animal trypanosomiasis, depending on availability, trypanosome and host species.

Compounding this challenge is the lack of a coordinated human–animal trypanosomiasis surveillance, pharmacovigilance programs, or funding for the development of veterinary trypanocidal drugs. The new human drugs, fexinidazole and acoziborole, have not been tested in veterinary models of trypanosomiasis, leaving AAT disease control reliant on outdated chemotherapies. This veterinary blind spot threatens progress toward HAT elimination. This is even more imperative and urgent considering that the World Health Organisation (WHO) has set the goal to eliminate gambiense HAT as a public health problem by the year 2030 [217,218].

#### 3.3.4. Current Developments Towards New Veterinary and Human Trypanocides

**Figure 10:**
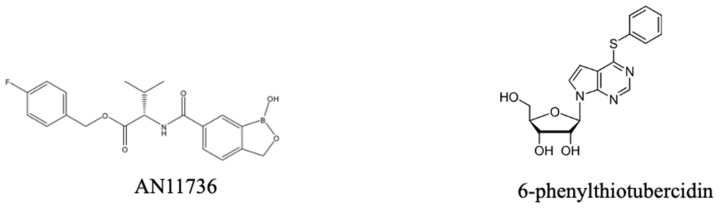
Important leads for new veterinary trypanocides. The figure highlights emerging lead compounds for animal African trypanosomiasis (AAT), the benzoxaborole AN11736 and the purine nucleoside 6-phenylthiotubercidin. These novel scaffolds exhibit potent trypanocidal activity and represent promising alternatives to legacy trypanocides. Their chemical structures underscore innovative mechanisms of action, supporting continued preclinical evaluation to establish efficacy, safety, and suitability for livestock field use.

One important lead for a new veterinary trypanocide is a benzoxaborole, AN11736 (Figure 10), which showed sub-nanomolar or very low nanomolar efficacy against *T. brucei*, *T. congolense* and *T. vivax* in vitro and 100% cure in mouse models with a single i.p. dose of 10 mg/kg [219,220]. It would be the veterinary equivalent of acoziborole, not just because it is of the same chemical class, but also because it has the same intracellular target, CPSF3 [178], which is also the target of benzoxaboroles in apicomplexan parasites [221,222]. AN11736 was found to be a prodrug that is activated by the parasite’s serine carboxypeptidases (CBPs); the cleavage by the peptide also drives the uptake of the drug into the cell by maintaining the concentration gradient of the prodrug across the plasma membrane [220]. *T. b. brucei* and *T. congolense* strains adapted to AN11736 resistance in vitro displayed mutations in the CBP locus, leading to a failure to activate the prodrug. There was no cross-resistance to acoziborole as this molecule does not contain the peptide–bond linker upon which the peptidase acts [220]. It would be expected, however, that any resistance mutations in the benzoxaborole target, CPSF3, would lead to cross-resistance to the entire class. Nonetheless, this compound does show substantial promise, especially if the one-dose cure translates into larger domestic animals, notably dogs, pigs, goats and cattle.

A second promising class of veterinary trypanocides is the nucleoside analogues. Nucleoside antibiotics such as tubercidin (7-deazaadenosine) and cordycepin (3′-deoxyadenosine) have long been known to have potent trypanocidal activity but tubercidin is too toxic and cordycepin is metabolically unstable, being a substrate of human adenosine deaminase [223]. While the stability of cordycepin could be rescued by halogenation on position two of the purine ring, this also resulted in a less active compound [224]. However, the combination of the tubercidin and cordycepin motifs (3′-deoxy-7-deazaadenosine; 3′dTub) yielded a much less toxic compound that was not a substrate for human adenosine deaminase. This compound displayed nanomolar EC_50_ values to *T. brucei* and sub-nanomolar EC_50_ against *T. b. rhodesiense* in vitro; the 7-bromosubstituted analogue was even more potent but less tolerated in vivo. 3′dTub was curative in mouse models of even late-stage (CNS) trypanosomiasis using oral administration [225].

The main limitation of the 3′dTub series, however, is that the compounds require uptake mediated by the TbAT1/P2 transporter as they are poor substrates of the related P1 adenosine carriers, which would make them cross-resistant with the important diamidine and arsenical drug classes [226,227,228] and ensure low activity against *T. congolense*, which lacks a P2 orthologue [229]. In contrast, a series of 7-aryl substituted tubercidin analogues (with intact ribosides) was equally well taken up through both transport systems, avoiding the cross-resistance [225]. Recently, the P1 adenosine transporters of the main animal trypanosomiasis pathogens have been cloned and characterised with a full structure–activity analysis, and adenosine analogues with EC_50_ values below 0.1 µM for all relevant species were identified [230]. This has culminated in the discovery of the most promising nucleoside trypanocide to date, 6-phenylthiotubercidin, which displayed nanomolar EC_50_s against all relevant parasite species in vitro and, for the first time, was fully curative in a mouse model of *T. vivax* as well as other African trypanosome species. The compound was metabolically stable and P1 adenosine transporter substrate, circumventing issues with cross-resistance. The compound did not display any genotoxicity and relatively low ecotoxicity versus aquatic organisms [49].

**Figure 11:**
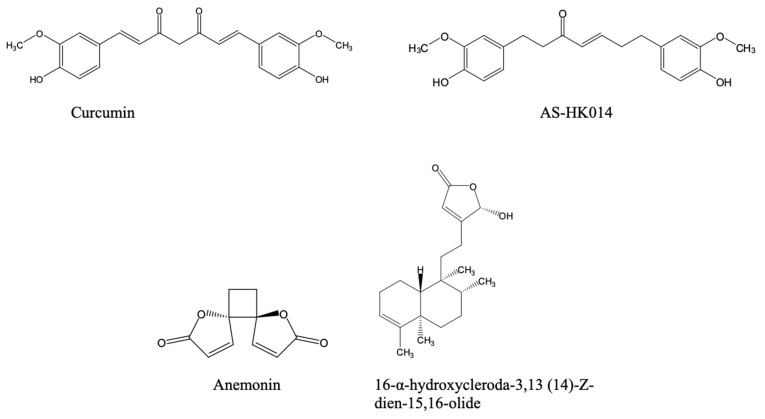
Natural compounds in preclinical development against AAT and HAT. The figure presents selected natural products showing activity against animal and human African trypanosomiasis, including curcumin (a polyphenolic diarylheptanoid), AS-HK014 (a monoketo analogue of curcumin), anemonin (a plant-derived lactone), and 16-α-hydroxycleroda-3,13(14)-Z-dien-15,16-olide (a clerodane diterpenoid). These diverse chemical classes highlight the potential of natural scaffolds as preclinical leads for developing safer and more effective trypanocidal therapies.

There are also several natural compounds at various stages of preclinical developments against AAT and/or HAT (see Figure 11). Curcuminoids, for example, have been evaluated against bloodstream forms of *T. b. brucei* (a rodent-infective surrogate frequently used preclinically for human HAT but equally useful as a model for surra and dourine), where structure–activity work around curcumin, including dibenzylideneacetone analogues, revealed sub-micromolar potency but flagged challenges in stability and ADME that warrant medicinal chemistry optimisation [231]. Curcumin itself is widely used as a spice, health food supplement and traditional medicine [232] and has been shown to have low micromolar activity against *T. brucei* in vitro [123,231]. A monoketo analogue of curcumin, 1,7-bis(4-hydroxy-3-methoxyphenyl) hept-4-en-3-one (AS-HK014), showed mid-nanomolar activity against wild-type and multi-drug-resistant *T. brucei* [123]. Extensive mechanism studies including metabolomics revealed that AS-HK014 made an adduct with trypanothione [233]. One important observation from this work was that curcuminoids are generally not cross-resistant with diamidines and arsenical trypanocides [123]; indeed, with our considerable experience in screening natural compounds against trypanosomatids, we have yet to document any cross-resistance between plant-derived chemicals and the chemical trypanocides. Moreover, resistance to curcumin itself could not be induced in vitro despite very considerable efforts [233].

For veterinary parasites, anemonin—a lactone isolated from *Ranunculus multifidus*, a plant used ethnoveterinarily in Ethiopia—exhibited potent in vitro and in vivo activity against a field isolate of *T. congolense*, clearing parasitaemia in mice and outperforming diminazene in relapse prevention [234]. This is a rare example of a plant metabolite that was directly advanced against AAT-relevant species and underscores the value of ethnoveterinary pipelines for livestock health and rural livelihoods.

The ethnopharmacological approach also identified a series of compounds from *Polyalthia longifolia*, the most promising of which was a diterpenoid, 16-α-hydroxycleroda-3,13 (14)-Z-dien-15,16-olide, with sub-micromolar activity against *T. brucei*, and multiple drug-resistant strains [235] with a multi-target mode of action [236].

Reviews of African and Nigerian flora catalogue additional antitrypanosomal candidates (e.g., iridoids from *Morinda lucida*, alkaloids from *Holarrhena Africana*, and *Monodora myristica*) with activity against *T. b. brucei* or *T. congolense* in rodent models, reinforcing the breadth of tractable natural scaffolds for veterinary indications [237,238,239].

Another rich source of anti-kinetoplastid compounds is propolis, a product bees make from plant resins to coat their hives and, apparently, self-medicate against parasite infection [240,241]. Compounds isolated from propolis from across the globe (e.g., Libya [242], Papua New Guinea [243], Europe [244], and Nigeria [245]) show great promise against a range of trypanosomatids, particularly veterinary trypanosomes. The widespread availability of bee propolis and its non-toxicity to humans make it an excellent starting point for locally produced One Health antiparasitics [246].

From a One Health standpoint, these natural product leads invite dual-use programs: compounds validated against *T. congolense* or *T. vivax* can be co-developed for HAT surrogates, while agricultural formulations (e.g., long-acting injectables) can be engineered alongside oral human dosage forms. Such co-development would help reduce livestock infection pressure that sustains tsetse transmission cycles. Additionally, it can be considered that drugs specifically developed for and applied to surra, dourine and other forms of non-tsetse-transmitted trypanosomiasis are unlikely to impact human treatments as the causative species are not human-infective.

## 4. Comparative Analysis of Anti-Trypanosomatid Drugs for Human vs. Veterinary Diseases: A One-Hundred-Year-Old Trend

Table 1 provides a list of pharmaceutical agents used for treating human kinetoplastid diseases, including key characteristics and considerations. This table summarises 14 drugs used for the treatment of human leishmaniasis, Chagas disease, and HAT. For each agent, the approval year, target parasites, mechanism of drug action and resistance are presented. Among the mechanisms of action are interference with nucleic acid synthesis, membrane disruption, enzymatic inhibition, and organelle dysfunction. Mechanisms of action (MOA) and mechanisms of resistance (MOR) are drawn largely from published laboratory research, and it is not always clear whether these mirror the MOA and MOR in the field.

Veterinary drugs used for the treatment of kinetoplastid infections in animals are listed in Table 2. These include human drugs adapted for treating the veterinary forms of the disease. While reports on drug resistance in the animal form of these diseases are rare, and reports are sometimes extrapolated from human research, this table presents key pharmacological features of veterinary agents used in managing animal African trypanosomiasis and canine leishmaniasis. Each drug is listed alongside its year of approval, host species (e.g., cattle, goats, dogs), mechanism of action, and known resistance concerns. Mechanisms include interference with parasite DNA, mitochondrial function, and purine metabolism. Resistance concerns are based on regional reports of decreased efficacy or confirmed resistance mutations in target parasite populations. The table highlights the urgent need for veterinary-specific drug innovation within a One Health framework.

A comparative analysis of registered pharmaceuticals for human and veterinary kinetoplastid diseases (Table 1 and Table 2) reveals a stark imbalance in drug development and regulatory investment. As shown in Table 1, the human pharmacopeia includes 14 drugs spanning multiple chemical classes and mechanisms of action developed over the past century. These agents target diverse biochemical pathways such as DNA damage and oxidative stress (e.g., nifurtimox, benznidazole, and fexinidazole), sterol biosynthesis (e.g., posaconazole), protein translation (e.g., paromomycin), and RNA processing (e.g., acoziborole). Despite their varying efficacy and toxicity profiles, this diversity underscores a relatively consistent, albeit historically delayed, effort to innovate within the human-health domain.

In contrast, the veterinary pharmacopeia is restricted to only a few registered drugs, most of which were introduced decades ago and have undergone minimal structural or formulation refinement. These include diamidines (diminazene aceturate), phenanthridines (isometamidium chloride and homidium bromide), and antimonials (e.g., meglumine antimoniate), with mechanisms largely centred on DNA binding and mitochondrial disruption. There are no veterinary drugs currently targeting kinetoplastid-specific RNA processing enzymes or sterol biosynthesis pathways, highlighting the translational gap from human to veterinary settings. Furthermore, widespread resistance to these limited agents, especially in *T. congolense* and *L. infantum*, which has been documented in multiple endemic regions, is compounded by inconsistent dosing practices and poor pharmacovigilance [5,224,233].

Despite the WHO recommendation not to use antileishmanial drugs used for treating the human forms for veterinary purposes, due to the potential of drug resistance development [254], these drugs are still often used for treating dogs with canine leishmaniasis, either as monotherapy or, for canine leishmaniasis, in various combinations. While all the currently available chemotherapies used in the treatment of canine leishmaniasis can induce sustained or temporary remission of clinical symptoms, none can achieve complete parasitological cure, leaving the animal a parasite carrier for potential transmission. Essentially, these therapeutics were originally developed to treat human leishmaniasis and afterwards repurposed for veterinary application. Consequently, most of the treatment procedures in dogs were adapted from human clinical protocols, often without sufficient pharmacodynamic and pharmacokinetic data that are specific to canine physiology. This signifies a critical gap in One-Health-aligned drug development, where veterinary-specific considerations remain underexplored despite dogs playing a central role as zoonotic reservoirs in leishmaniasis disease transmission. Although allopurinol is now considered a drug for veterinary leishmanioses only, it was originally trialled for various forms of human leishmaniasis, including in combinations, and is still being used in human infection, for instance, in combination with pentavalent antimony [143] or in combination with pentamidine against Sb(V)-unresponsive VL [255]. In fact, allopurinol has also been trialled for Chagas disease (but not animal infections with *T. cruzi*) [256] and reportedly acts synergistically with benznidazole in mice [257].

For AAT, the situation is slightly different, in that phenanthridines and quinapyramine were specifically developed as veterinary drugs and, to the best of our knowledge, never used in humans, and are certainly not licenced to be. Diminazene aceturate was developed as a veterinary equivalent of the diamidine pentamidine, which has remained almost exclusively for use against HAT. Similarly, melarsomine is the veterinary equivalent of melarsoprol, of which it is a more soluble derivative; it is not licensed for human use. For the veterinary equivalent of Chagas disease, as we have seen, there is no licenced or suggested chemotherapy at all.

**Figure 12:**
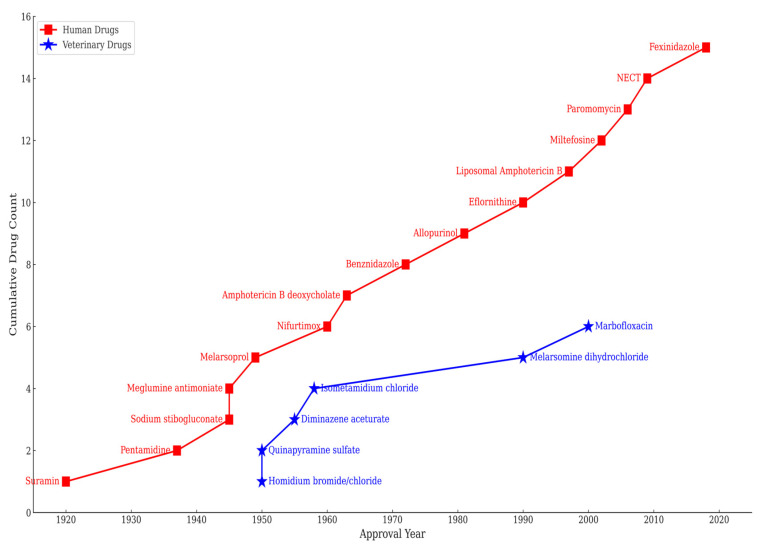
One-hundred-year-old trend of registered drugs for kinetoplastid disease. The figure compares the timeline of drugs used in treating kinetoplastid diseases: human vs. veterinary-only drugs. It shows a graph of drug index (the cumulative number of approved drugs) against the corresponding approval year, which is the approximate year in which the drug was first used for human or animal trypanosomatid infection. The veterinary-only drugs are drugs specifically approved for use in animal trypanosomatid infections. For context, human drugs adopted for veterinary use were omitted in the veterinary-only drug trend.

Figure 12 starkly demonstrates the historical one-sidedness in drug development for treating diseases caused by kinetoplastid parasites. The figure reveals a clear tilt towards human-centric therapeutics, showing that veterinary-specific drug discovery remains largely underdeveloped and that no new agents have been introduced in the last 25 years. For AAT, the most-used drugs were all introduced before 1960. Despite the zoonotic and ecological interconnection of all three of these diseases, veterinary-only drug approvals, be they therapeutic or prophylactic—remain scarce and stagnant.

As can be seen in Figure 1, most of the veterinary-only drugs were introduced over half a century ago. In the context of disease control and One Health, the absence of any prophylactic therapeutics except the old trypanocide isometamidium, for which resistance is widespread [5], is particularly glaring. In contrast, human-focused drug discovery, though still limited, shows more recent momentum. This lopsided investment in anti-trypanosomatid drug development undermines disease control efforts and, as we discussed above, this imbalance continues in current drug development efforts.

The neglect of animal reservoirs, with infections either not treated or treated sub optimally, almost ensures the continued need for new human antiparasitics, as the situation propagates the disease transmission cycles. From a One Health perspective, the nonexistence of a well-coordinated pharmaceutical development plan approach across species signifies a missed opportunity to control the disease burden more holistically and highlights the critical demand for integrated research and development pipelines that exceed the present human-centric paradigms.

This disparity is emblematic of a broader structural failure to integrate animal health into drug development pipelines despite the zoonotic and ecological entanglement of kinetoplastid disease transmission. It reflects a siloed approach that prioritises human therapeutics while neglecting the animal reservoirs that perpetuate transmission cycles. The absence of co-developed, dual-indication drugs, particularly for species such as dogs (in canine visceral leishmaniasis) and livestock (in AAT), limits the feasibility of One-Health-aligned disease elimination strategies. (Note: although we do not discuss it here, we are aware that cats are increasingly reported with clinical leishmaniosis and should be considered an additional reservoir host [258].)

To bridge this divide, future drug pipelines must explicitly incorporate veterinary target product profiles (TPPs), host-specific pharmacokinetics, and species-appropriate delivery platforms from early stages of development. Harmonised regulatory frameworks and cross-sectoral funding mechanisms are essential to incentivise the creation of safe, efficacious, and field-adapted drugs for both humans and animals. Such integrative pharmaceutical stewardship is central to achieving sustainable control of kinetoplastid diseases and realising the full potential of the One Health paradigm.

## 5. Control Strategies Other than Chemotherapy

Kinetoplastid diseases are closely tied to complex ecological and socioeconomic systems, involving interactions among humans, animals, and environmental health [31]. An effective and sustainable control approach requires not only advancements in chemotherapy alone, but also the incorporation of a robust vector control strategy entrenched within an integrated One Health approach [259]. Computational models by [31] clearly show that especially for rhodesiense but also for gambiense HAT, vector control by insecticide is more effective than a chemotherapeutic approach [31]. Yet, more attention is currently given to chemopreventive or treatment strategies using trypanocides. Consequently, there is more relative progress in chemotherapy, at least as regards human infection, than in vector control. Chemoprophylactic treatments such as isometamidium for AAT can reduce transmission but need close monitoring to avoid the appearance of drug-resistant strains of trypanosomes [260]. Vector control strategies include vegetation clearing, insecticide application, and insecticide-treated livestock. Other measures include public health interventions such as active screening campaigns, protective clothing, insecticide-treated bed nets, and awareness and education [261]. This section, therefore, evaluates the evidence supporting vector control as a fundamental pillar in the management of these vector-borne kinetoplastid diseases. It examines some historical successes, operational challenges, and potential opportunities.

### 5.1. African Trypanosomiasis: Suppressing Transmission Through Tsetse Control

Integrated tsetse control programmes that combine various methods are fundamental to interrupting transmission of trypanosomiasis by reducing vector density. The control of African trypanosomiasis, especially the gambiense and rhodesiense forms, has shown the potential of targeted vector management. One of the most compelling pieces of evidence of successful vector control is the eradication of HAT from Unguja Island (Zanzibar) in the 1990s using the Sterile Insect Technique (SIT) [251,252]. Through the release of sterile male *Glossina austeni*, local tsetse fly populations were eradicated, effectively interrupting disease transmission [262]. One of the major advantages of using the SIT strategy is that its environmental impact is much less consequential than that of insecticide aerial spraying of infested areas. The SIT programme, therefore, highlights the feasibility of sustainable area-wide integrated pest management when implemented but requires sustained financial and political commitment. However, sustained eradication of tsetse on mainland Africa may be much harder to achieve by SIT except in highly isolated foci because of the risk of re-infestation from neighbouring areas and the presence of multiple species of tsetse in many areas.

In regions of East Africa, especially those bordering the wildlife conservation areas, pour-on insecticide-treated cattle have drastically reduced tsetse populations, resulting in a corresponding decrease in the incidence of AAT [263]. In this method, synthetic pyrethroids that are stable and persistent in the field, with low toxicity to mammals but lethal to tsetse flies, are applied directly onto the cattle’s skin, killing the fly when it lands on the treated animal for a blood meal [264]. This approach offers the advantage of cost effectiveness and practicality to controlling tsetse and AAT, particularly when compared to other methods like the trap and target approach, whose deployment and maintenance are faced with the challenge of the usually large, often unreachable terrains, and the scientifically difficult approach to the design of the control programme, which require some level of organisation and infrastructure rarely available in endemic regions [265]. In the One Health context, the use of general (pyrethroid) insecticides at a large scale is disfavoured because of its ecotoxicity but these issues can be mitigated by restricting insecticides to the most high-risk tsetse-infested sites, to the most attractive hosts, and indeed to the preferred feeding sites on those hosts, e.g., the lower legs of cattle. Bouyer et al. (2009) reported very significant reductions in bovine trypanosomiasis in Burkina Faso by using insecticide footbaths [261]. This strategy has the significant extra advantage of killing other insects and ticks feeding on the animals [266].

These veterinary interventions, if implemented, present a scalable and sustainable solution for high-risk areas and would contribute to the reduction in the incidence of rhodesiense HAT infections in human populations, especially those sharing (or adjacent to) ecosystems with livestock reservoirs.

### 5.2. Vector Control and Chagas Disease

The control of Chagas disease in Latin America remains one of the most extraordinary public health achievements in vector-borne disease management. Key to this achievement is the Southern Cone Initiative (SCI). It was formally launched in 1991 by the Southern Cone countries of Argentina, Brazil, Bolivia, Paraguay, Uruguay, and Chile) [258,259]. The SCI was a comprehensive regional effort targeting *T. cruzi* transmission through coordinated strategies that combined transfusion safety and vector elimination [267]. The SCI’s primary goals were (i) to eradicate domestic and peridomestic populations of *Triatoma infestans*, which is the main vector; (ii) to reduce infestations by other triatomine species in overlapping endemic zones; and (iii) to eliminate transfusion-based transmission of the parasite via systematic blood donor screening [268] and/or treatment of the donated blood [269].

The SCI’s success was reliant on a two-phase approach. (i) The “treatment phase” involved indoor residual spraying (IRS) of homes using synthetic pyrethroids such as cyfluthrin, lambda-cyhalothrin, or deltamethrin, conducted by experts. (ii) The “surveillance phase” involved a community-based surveillance system where householders were asked to report vector sightings, which then triggered targeted retreatment [260,263].

The programme produced impressive outcomes. *T. infestans* was largely eliminated from Brazil, Chile, Uruguay, and large parts of Argentina, considerably reducing vectorial transmission of *T. cruzi* [270]. The success also underscored the significance of incorporating housing improvement programs to remove sanctuaries for triatomine bugs [271], thereby strengthening physical and ecological barriers to disease transmission. The strategy involved the replacement of abode and thatch dwellings, which were commonly infested [258,260].

Inspired by the Southern Cone’s success, in 1997, the initiatives of Central America and Mexico (IPCAM), of the Andean Countries (IPA) and of the Amazon countries (AMCHA) were created. Interestingly, all the aims of these initiatives were similar to those of the SCI [261,263,265]. For the Andean countries’ initiatives, Venezuela, in particular, has sustained one of Latin America’s longest-running national Chagas control programmes, although its success has received relatively little scholarly attention [268]. The Venezuelan experience, in terms of sustained vector surveillance and community education, provides profound insight for sustainability and regional adaptation.

Also, blood donor screening, a complementary non-vector intervention, has been vital in reducing iatrogenic transmission. Since the 1990s, rigorous screening protocols have been implemented across Latin America [272]. This strategy drastically reduced transfusion-related cases. Although not a vector control measure in itself, this approach demonstrates the effectiveness of multidimensional, integrated intervention in curbing disease spread. Further measures that can be implemented are reductions in vertical transmission by antenatal screening and in food-borne transmission by better regulation and practice in food production [272]. In the case of Chagas disease, it is unquestionable that, to date, non-pharmaceutical control measures have had far more impact than chemotherapy. The development of new, more effective human and veterinary drugs combined with earlier detection has the potential to change that equation.

### 5.3. Integrating Vector Control with Pharmaceutical Intervention in Leishmaniasis Management

While considerable progress has been made in antileishmanial drug discovery, integrating vector control into public health strategies is essential for attaining enduring disease control. Leishmaniasis and phlebotomine sandflies thrive in ecological niches where vector proliferation is unchecked. Therefore, curbing vector populations directly complements therapeutic interventions by reducing the intensity of transmission and the need for treatment, particularly in endemic areas.

There are instances where vector control was integrated with pharmaceutical interventions. For example, in India, the National Kala-azar Elimination Programme made vector control, specifically Indoor Residual Spraying (IRS), a cornerstone of its disease control strategy [273]. Using synthetic pyrethroids such as alpha-cypermethrin, IRS was vital in reducing *Phlebotomus argentipes*—the main vector of visceral leishmaniasis in the region [27,267]. Studies have shown that biannual IRS, when deployed with high community coverage, drastically reduces *Phlebotomus* densities with a corresponding decrease in the incidence of visceral leishmaniasis [87,266,267]. The combination of early diagnosis, effective treatment (e.g., with liposomal amphotericin B), monitoring for pyrethroid-resistant sandflies, and sustained IRS brought India to the brink of eliminating visceral leishmaniasis as a public health problem [27,266,268] at a time when chemotherapeutic strategies were severely challenged by drug resistance [274].

In Brazil, environmental management, insecticide-treated nets (ITNs), and canine reservoir control were integrated in vector control strategies [269,270], impacting both Chagas disease and leishmaniasis. These strategies resulted in variable but significant success [275]. ITNs reduce indoor vector–human contact, particularly in urban fringes and rural communities where sandfly breeding sites are abundant [56]. While their efficacy may be modest, ITNs offer additional protection when combined with chemotherapy, improved animal husbandry practices, and household education [275].

Another example of regional integrated efforts in leishmaniasis control can be seen in the East Africa vector control programme. East African countries like Sudan, Ethiopia, Uganda, and Kenya have incorporated the Integrated Vector Management (IVM) strategy in their WHO-supported frameworks [276]. These programmes deploy insecticide-treated nets, IRS, and community mobilisation programmes along with diagnostic and treatment initiatives to achieve its overall goal [277]. The goal is to reduce the incidence of visceral leishmaniasis by 90% by the year 2030. This is to be achieved through an organised regional effort [278].

Leishmaniasis disease burdens have increased in Georgia and other trans-Caucasian and Central Asian former Soviet republics since control measures from the Soviet era ended, providing an important example of vector control success followed by reversal [279]. In the Soviet era, IRS campaigns were used to control mosquitoes and malaria, but this strategy also curbed sandfly populations and subdued the transmission of leishmaniasis, which led to a near eradication of leishmaniasis in multiple Soviet republics, including Georgia [280], also aided by the elimination of stray and infected dogs [281]. However, after the disintegration of the Soviet Union, there was a cessation of vector control programmes in Georgia and most of the other newly independent republics, followed by a resurgence in sandfly populations, including *Phlebotomus balcanicus*, *P. kandelakii*, *P. halepensis* and *P. sergentii* [282], which reintroduced both the human and canine forms of leishmaniasis, which reached an alarming level, particularly in the Tbilisi area [35,279]. Numbers from a review of worldwide incidence of VL over the period of 2004–2008 show that Georgia had by far the highest reported and estimated number of VL cases in the former Soviet Union [283] but these are reported to have somewhat declined since their peak around 2005–2010 [284], although it must be stressed that the numbers are based on mostly passive case finding, leading to severe underreporting. The Georgia experience is a warning to other regions that have managed to eliminate vectors, highlighting the need for sustained, active surveillance programmes. Importantly, the high incidence of human VL in Georgia is almost certainly driven by very high prevalence in local domestic and stray dogs, reaching 28.1% and 26.9% in two out of the three Tbilisi districts surveyed using the rK39 rapid diagnostic test [35].

In addition, the rise in cases of cutaneous leishmaniasis and the spread of *L. tropica* and *L. donovani* in Turkey and Azerbaijan, despite pharmaceutical interventions, pose a growing threat to southern Europe, where phlebotomine sandflies are already established [284]. Tackling this regional human and animal health challenge requires putting in place effective diagnostic, therapeutic, and vector control measures.

The case studies discussed in this section show that pharmaceutical intervention alone is not enough to achieve enduring disease control. Rather, integration of vector surveillance, insecticide resistance monitoring, environmental sanitation, and public education will provide a collaborative framework that will advance the efficacy of therapeutic interventions and limit the transmission of disease in the long term.

## 6. Perspectives Toward a One-Health-Aligned Anti-Trypanosomatid Drug Development Strategies

In this section, we discuss a wide-ranging roadmap for transforming drug development for kinetoplastid diseases through the lens of One Health by positioning human, animal, and environmental health priorities across every phase, from target discovery to real-world access. These approaches aim to operationalise a cross-species, sustainable, and ecosystem-aware pharmaceutical transformation.

### 6.1. Holistic Target Prioritisation Across Host and Vector Interfaces

Kinetoplastid parasites exploit conserved metabolic pathways such as sterol biosynthesis, CPSF3-mediated RNA processing, and trypanothione reductase, which are important across parasite species and lifecycle stages [61,157,282]. Highlighting these pan-kinetoplastid targets facilitates the development of agents with a broad spectrum of activity across *T. brucei*, *T. cruzi*, and *Leishmania* in both insect and mammalian hosts. Including vector-stage bioassays in early compound screening, such as assays targeting tsetse fly or sandfly, may facilitate the discovery of transmission-blocking agents and enhance long-term epidemiological impact.

### 6.2. Co-Development for Human and Veterinary Indications

Historically, human and animal chemotherapy have evolved on parallel tracks, despite overlapping disease ecologies. For example, acoziborole, with potential to be used as an oral single-dose drug with durable action [285], can be repurposed for reservoir species if veterinary pharmacology and toxicology studies are incorporated in the initial drug discovery process. Species-specific formulations, such as long-acting injectables for cattle or chewables for dogs, should be designed in parallel with human-phase trials to streamline regulatory pathways and exploit strategic deployment in zoonotic hotspots.

### 6.3. Inclusion of Ecological Pharmacology in Preclinical Development

The current traditional drug pipelines often overlook environmental consequences [286]. A One Health model demands preclinical analysis of ecotoxicological outcomes at the advanced lead compound stage. This includes bioaccumulation in non-target organisms, persistence of drug metabolites in the soil and water, and sublethal exposure of vectors via treated hosts [287]. Regulatory frameworks should therefore mandate drug residue analysis in excreta and environmental media, together with assays for effects on vector competence and microbiomes [288]. These principles should guide the prioritisation of development candidates and procurement policies.

### 6.4. Harmonisation of Human and Veterinary Regulatory Frameworks

The current regulatory framework is fragmented, and as such, not conducive to the development of new dual-use pharmaceuticals. Harmonising regulatory requirements through cross-species dossiers, streamlined co-registration, and shared pharmacovigilance will foster One Health drug development [287,288]. The quadruple (Quadripartite) agency initiatives by the WHO, WOAH, FAO, and UNEP [289] should establish a regulatory convergence paradigm that supports an integrated product approval and surveillance. Also, regional regulators like the African Medicines Agency (AMA), European Medicines Agency (EMA), and American Medical Association (AMA) can champion joint reviews to minimise duplication of function and fast-track market registration.

### 6.5. Local Manufacturing, Delivery Innovation, and Access Equity

A robust, quality-controlled local production capacity and context-adapted delivery models are vital to ensure equitable drug access. For instance, heat-stable, orally dosed chemotherapies will simplify the deployment of drugs for both humans and animals [290], especially in remote areas with reduced access to medical facilities. In veterinary settings, simple delivery platforms like injectables, chewables, or topical formulations must match field realities and align with herd health management. Accessing equity depends on market-shaping approaches such as tiered pricing, pooled procurement, and donor incentives tied to One Health drug impact [291].

### 6.6. Reframing Drug Development as a Transdisciplinary One Health Enterprise

One Health drug discovery requires a transdisciplinary collaboration [292] involving chemists, veterinarians, entomologists, ecologists, social scientists, and community stakeholders from the inception of a project. Grant mechanisms should incentivise cross-sector proposals that clearly address zoonotic and eco-health outcomes beyond human clinical efficacy [293]. Partnerships cutting across public–private domains should co-design drug pipeline solutions that are responsive to the global south stakeholders and ecosystem realities.

## 7. Conclusions

The kinetoplastid diseases, African trypanosomiasis, Chagas disease, and leishmaniasis, remain a persistent global health challenge despite being biologically well characterised and geographically well defined. Their sustained prevalence, morbidity, and socioeconomic impact are a direct consequence of systemic failures in drug development, access, and integration. Notwithstanding the approval of some new drugs, notably fexinidazole for HAT, treatment and control continue to be hampered by various issues such as drug resistance, toxicity, limited spectrum of activity, and poor applicability to real-world endemic settings. From a veterinary perspective, the situation is even more dire, with outdated or counterfeit trypanocides, and a near-total lack of drug innovation for livestock trypanosomiasis, *T. cruzi* infection and animal leishmaniasis. These gaps facilitate cross-species transmission, undermine elimination campaigns, and hinder broader goals of health equity and sustainable development.

This review has shown that considerable advancement in pharmaceutical interventions for kinetoplastid diseases will require a fundamental reorganisation of how drug development is conceptualised, funded, delivered, and regulated. The traditional gap between human and veterinary medicine stands distanced from to the ecological realities of zoonotic and vector-borne diseases. A shift toward a One-Health-oriented model, i.e., one that integrates human, animal, and environmental aspects at every stage of the drug discovery lifecycle, is no longer optional. It is a strategic necessity.

To operationalise this vision, future pharmaceutical research and development must ensure the co-development of dual-use drugs or at a minimum ensure the timely development of both human and veterinary drugs, integrate environmental pharmacology into preclinical workflows, strengthen regional manufacturing and delivery systems, and harmonise regulatory frameworks across species. Multi-sectoral research groups must become the norm rather than the exception, and funders must recalibrate their investment schemes to reflect the interdependence of health systems.

Specific priorities for future work include the following:

- Expansion of drug target discovery programmes to include pan-kinetoplastid pathways that are amenable to cross-species pharmacology.
- Systematic pharmacokinetic and efficacy studies of new compounds in reservoir hosts.
- Evaluation of the impacts of drugs on vector infectivity and parasite development through transmission-blocking studies.
- Development of field-friendly, heat-stable formulations for both humans and animals.
- Establishment of a joint human–veterinary regulatory review process and post-market pharmacovigilance systems.
- Creation of an integrated access and stewardship programme that safeguards efficacy while ensuring availability and affordability across health sectors.

We conclude by noting that the future of kinetoplastid disease control depends not on isolated breakthroughs but on convergent innovation. This involves linking scientific discovery with ecological responsibility, social accountability, and regulatory foresight. Only through a coordinated, One-Health-aligned pharmaceutical approach can we expect to break the cycle of global disease burden, and protect the health of humans, animals, and the ecosystems they share.

## Figures and Tables

**Table 1:** Drugs used for treating human kinetoplastid diseases. Year of first use is the year of first approval for use against the indicated trypanosomatid infection.

Drug Name	Year of First Use	Target Parasite/ Disease Form	Mechanism of Action	Mechanism of Resistance	Side Effects/Major Limitations	Reference
Pentamidine	1937	*T. brucei*—effective against first stage *T. b. gambiense* infection. Though relatively less effective, it is also used in leishmaniasis, particularly the cutaneous form.	Not fully understood, but the drug is reported to bind to parasite DNA, inhibiting type II topoisomerase, and disrupting mitochondrial DNA.	Via reduced drug uptake due to alterations in transmembrane pentamidine transport proteins, specifically the TbAT1 adenosine transporter and the aquaglyceroporin TbAQP2.	Serious toxicity, including hypotension, hypoglycaemia, drug resistance. It is only used for treating stage 1 HAT as it does not permeate the BBB.	[58,65,207,247,248]
Suramin	1920	*T. brucei*—effective against first stage *T. b. rhodesiense*	Unclear but reported to be via induced collapse of cellular ATP which ultimately leads to trypanosome cell death. Also, it inhibits glycolysis enzymes, and non-specifically binds to L-α- glycerophosphate oxidase.	Unclear but linked to changes in variant surface glycoprotein, which confers resistance by binding suramin and potentially altering its uptake pathway. Mutations in a DNA helicase of *T. brucei* (RuvBL1) are reported to reduce suramin’s effectiveness.	Severe toxicity issues—nephrotoxicity, allergic reactions. It is highly polar and does not cross the BBB, meaning it can only be used for treating stage 1 HAT. It has a short half-life.	[208,249,250]
Melarsoprol	1949	*T. brucei*—first-line treatment for second stage *T. rhodesiense* HAT	Disruption of redox balance via inhibition of trypanothione reductase.	Mutations in transporters AQP2 and TbAT1.	Highly toxic with a narrow therapeutic window, drug counterfeiting, and prolonged treatment regimens. Reactive encephalopathy frequently occurs. Can cross the BBB, has a long half-life of 35 h and has been widely used for treating the late stages of HAT, although the accompanying encephalopathy and widespread drug resistance limit its use.	[18,206,251,252]
Eflornithine	1990	Meningoencephalitic (Second) stage *T. b. gambiense*.	Ornithine analogue that inhibits ornithine decarboxylase.	Deletion or mutation of the TbAAT6 gene in trypanosomes.	Has a short half-life; hence, a high dose (400 mg/kg/day) is needed. The i.v. regimen is also complex and cumbersome to apply. Note: Eflornithine is trypanostatic and not trypanocidal; so, it might not be effective in immunosuppressed patients.	[209,253]
NECT Nifurtimox/Eflornithine Combination Therapy	2009	*T. brucei*—Stage 2 *T. b. gambiense* infection	Combination of DNA damage and polyamine synthesis inhibition	Reduced activity of the enzyme *T. brucei* type I nitroreductase, crucial for activating nifurtimox. Also, reduced uptake of eflornithine, or mutations in the drug target.	It is not effective against *T. b. rhodesiense*. Requires inpatient care, trained personnel, and sterile supplies.	[210]
Fexinidazole	2018	*T. brucei*—First and non-severe second stage *T. b. gambiense* and *T. b. rhodesiense*	Activation via nitroreductases causes free radical damage selective damage of the parasites’ DNA and proteins.	Similar to Nifurtimox. Loss of activity of a type I nitroreductase (NTR) enzyme via gene deletion or decreased transcription.	Decreased cure rates and unacceptable side effects in patients with severe stage 2 HAT. At the time of writing, safety and efficacy studies have not been established in children < 6 years or weighing <20 kg.	[180,181,207]
Benznidazole	1972	*T. cruzi*	Nitroreductase enzyme mediated DNA damage and oxidative stress from free radicals.	Mutations in the nitroreductase (TcNTR-1) gene, changes in chromosome copy number, and activation of other genes involved in drug resistance.	Several unwanted side effects have been reported, including abdominal pain, rash or skin lesions, weight loss, nausea, vomiting, diarrhoea, dizziness, and tremor. Potentially teratogenic—cannot be used by pregnant women or women who may become pregnant.	[143,208,209]
Nifurtimox	1960	*T. cruzi*	See benznidazole.	See benznidazole.	Several, including decreased appetite, diarrhoea, dizziness, fever, headache, nausea, stomach pain, vomiting, and weight loss. Highly toxic, existence of drug counterfeiting, and prolonged treatment regimens. Potentially teratogenic—cannot be used by pregnant women or women who may become pregnant.	[18,210,211]
Miltefosine	2002	*Leishmania* species	Interferes with phospholipid metabolism, disrupts Ca^2+^ homeostasis, and inhibits mitochondrial cytochrome C oxidase.	Aminophospholipid transporter gene mutation linked with decreased drug uptake, or increased drug efflux via overexpressed ABC transporters. Also, suppression of oxidative stress-induced programmed cell death.	Adverse effects include renal toxicity and gastrointestinal disturbances, but these symptoms are reversible. Teratogenic. Susceptibility varies by *Leishmania* species and geographic regions.	[58,101,212,213]
Amphotericin B deoxycholate	1963	*Leishmania* species	Binds to (leishmania-specific) ergosterol, and forms membrane pores.	Alterations in the parasite’s sterol composition and membrane properties.	Reports of serious adverse reactions, including fever with rigor and chills, renal dysfunction, severe hypokalaemia, occasional serious myocarditis and death. Its use requires close monitoring and prolonged hospitalisation.	[84,88,89,214,215]
Liposomal Amphotericin B	1997	*Leishmania* species	See Amphotericin B deoxycholate.	See Amphotericin B deoxycholate.	In the lipid formulations (AmBisome), deoxycholate is replaced with other lipids that mask Amphotericin B. AmBisome is considered relatively non-toxic and highly effective form of treatment for VL. The need for cold-chain storage is a challenge in rural areas.	[88,89]
Sodium stibogluconate	1945	*Leishmania* species	Reduces to Sb(III) and disrupts redox balance.	Changes in gene expression for antimony transport protein LdAQP1 and glutathione metabolism leading to decreased drug uptake, increased drug efflux, and changes in parasite metabolism.	Toxic, severe side effects, including anorexia, nausea/vomiting, abdominal pain, headache, raised liver enzymes, coughing and substernal pain, fever, sweating, flushing, bleeding from nose or gum, jaundice, and rash.	[61,62,65,216]
Meglumine antimoniate	1945	*Leishmania* species	The drug reduces to Sb(III) and disrupts redox balance by disrupting glutathione (GSH) (trypanothione).	Reduced drug uptake, increased drug efflux, and alterations in antioxidant defence mechanisms resulting in diminished biological reduction of Sbv to Sb (III).	Associated severe side effects and toxicity concerns, including local pain at the site of intramuscular injection, cardiotoxicity, hepatotoxicity, pancreatitis, and nephrotoxicity. Also, there are reports of emergence of resistance and therapeutic failures in certain regions.	[61,216,217]
Paromomycin	2006	*Leishmania species* especially *L. donovani*	Inhibits leishmania protein synthesis by binding to the ribosomal subunit and via depolarisation of mitochondrial membrane potential (MMP).	Decreased drug accumulation due to alterations in the parasite’s cell membrane composition.	Common side effect includes ototoxicity, and problems with liver function.	[58,78,80,218]

**Table 2:** Drugs used for treating animal kinetoplastid infections in domestic animals. This list includes human drugs adapted for treating the veterinary forms of the disease. Approval year is the year of first reported use for veterinary purpose.

Drug Name/Chemical Formula	Approval Year	Host Species	Target Parasite	Mechanism of Action	Mechanism of Resistance	Side effects & Other Treatment Issues	Reference
Diminazene aceturate	1955	Cattle, goats, sheep, pigs	*Trypanosoma* spp. (*vivax*, *evansi*, *congolense*, *brucei*)	Binds kinetoplast DNA, inhibits replication.	Reduced drug uptake by *T. brucei* due to mutations in transporter gene TbAT1. In *T. congolense*, reduced mitochondrial membrane potential (MMP).	Increase in body temperature (in sheep), decrease in body temperature (in cow): respiratory and heart rates increased (in sheep and horses), and decreased respiratory rates (in cows). Widespread resistance in multiple regions.	[5,197,219]
Isometamidium chloride	1958	Cattle, goats	*Trypanosoma* spp. (*vivax*, *evansi*, *congolense*)	Accumulates in mitochondria, disrupts kinetoplast.	Impaired drug uptake, reduced MMP and altered drug targets (kDNA).	Resistance is widespread in West Africa.	[26,220,221,222]
Homidium bromide/chloride	1950s	Equids	Widely used in the treatment of AAT caused by *Trypanosoma* spp. Including *T. vivax*, *T. evansi*, *T. congolense*. Low efficacy in *T. b. bucei* infection.	It disrupts the kinetoplast DNA (kDNA) network by intercalating parasite DNA, disrupting its structure and replication, leading to the loss of kDNA, and at high doses, inhibits nuclear DNA replication.	Mutations in genes encoding for drug transporters, resulting in reduced drug uptake, or alterations in drug targets.	Widespread drug resistance issues. The drug exists in powder form. It is highly carcinogenic. Administration is via IM; it causes toxic effects in horses.	[223,224]
Suramin sodium	1920s	Horses, camels, dogs	*Trypanosoma* spp. (*brucei*, *evansi*)	Linked to inhibition of energy metabolism by inhibiting enzymes involved in glycolysis, particularly in the glycosomes. Effects on the mitochondrial membrane potential.	Multiple, including loss of function of the suramin receptor (ISG75), endosomal proteins, lysosomal proteases (Cathepsin L), and lysosome-based major facilitator superfamily (MFST).	Rarely studied in treated animals and underreported.	[203,225,226]
Quinapyramine sulfate	1950s	Horses, camels, cattle	*Trypanosoma* spp. (*evansi*, *vivax*, *congolense*, *brucei*, *equiperdum*, *simiae*)	Interferes with parasite mitochondrial function, inhibits DNA and protein synthesis–potentially by displacing magnesium ions and polyamines from ribosomes. Causes morphological changes in the parasites–increased vacuolation of lysosomes and mitochondrial swelling.	Uncertain.	In equines, it causes salivation, diarrhoea, trembling, sometimes collapse in sensitive animals within minutes of treatment It is not frequently used in livestock, at least in Africa, because quinapyramine-resistant *T. congolense* displays cross-resistance to diminazene, homidium, and isometamidium.	[5,224,227,228,229]
Melarsomine dihydrochloride	1990s	Camels	*T. evansi*	Precise mechanism unknown but linked to binding thiol-containing enzymes and trypanothione; alters the parasite redox balance, and causes cellular damage, leading to parasite death.	Mutations in the adenine–adenosine transporter (P2/TbAT1), reduced uptake of the drug due to alterations in other membrane transport proteins, and increased expression of efflux pumps.	Clinical relapses can occur months to 1 or 2 years after treatment, more common with shorter duration of treatment (i.e., less than 4 weeks).	[224,230,231]
Meglumine antimoniate (*N*-methylglucamine antimoniate)	1996	Dogs	*L. infantum*	The drug reduces to Sb(III), and disrupts redox balance by disrupting glutathione (GSH) (trypanothione).	Reduced drug uptake via decreased Aquaglyceroporin-1 Expression. Increased detoxification via increase in the production of thiols, like glutathione, and enzymes involved in thiol metabolism, which can directly reduce Sb(V) to Sb(III), making it less toxic to the parasite.	Can cause pain and inflammation at the site of injection, potentially nephrotoxicosis, and rarely pancreatitis. Emerging resistance in endemic areas. Relapse or recrudescence can occur after 6–12 months of treatment.	[59,60,232]
Allopurinol	1980s	Dogs	*L. infantum*	Allopurinol is metabolised by the parasite into a toxic compound (4-amino-pyrazole-pyrimidine riboside monophosphate), which is incorporated into RNA, disrupting its structure and function.	Variations in gene copy numbers, including reduction in the S-adenosylmethionine synthetase (METK) gene.	Can cause nephrolithiasis, xanthine crystalluria, and urolithiasis. Recrudescence and relapse can occur after 4–6 months of treatment. Reduced efficacy due to chronic use.	[63,218,233,234]
Miltefosine	2000s	Dogs	*L. infantum*	Alters membrane lipid metabolism, induces apoptosis-like cell death, disrupts calcium homeostasis and inhibits cytochrome c oxidase.	Mutations in the miltefosine transporter (MT), a complex encoded by the MT and its regulatory subunit *ROS3* genes.	Causes vomiting, diarrhoea, and dysorexia. Recrudescence and relapse are frequent—can occur after 4–6 months. Widespread resistance in Latin America and Mediterranean. Unable to fully clear the parasite from infected dogs.	[235,236]
Paromomycin (Aminosidine)	2006	Dogs	*L. infantum*	Inhibitor of bacterial protein synthesis through irreversible binding to the 30S ribosomal subunit in mitochondria.	Associated with changes in membrane fluidity and increased expression of ABC transporters, which facilitate drug efflux.	Can cause ototoxicosis and nephrotoxicosis. Recrudescence or relapse can occur after 3–4 months.	[58,80,237]
Pentamidine	1980s	Dogs	*L. infantum*	Precise mode of action remains to be elucidated but acts on mitochondrial targets including the kinetoplast and affects the mitochondrial membrane potential.	Unclear, but likely due to reduced drug uptake and increased drug efflux.	Severe adverse effects: pain and necrosis at the injection site, diarrhoea, vomiting, systemic hypotension, hypersalivation, and anaphylactic shock	[235,238]
Marbofloxacin	2000s	Dogs	*L. infantum*	Unknown, but like other quinolones, it may selectively inhibit the enzyme DNA gyrase (or topoisomerase IV).	Mutations in the parasite’s DNA gyrase or topoisomerase II, the enzyme targeted by the drug.	Vomiting, decrease in appetite.	[239,240,241]

## Data Availability

No new data were created or analysed in this study. Data sharing is not applicable to this article.

## Abbreviations

The following abbreviations are used in this manuscript:

NTDs	Neglected tropical diseases
HAT	Human African trypanosomiasis
AAT	Animal African trypanosomiasis
DNDi	Drugs for neglected diseases initiative
kDNA	Kinetoplast deoxyribonucleic acid
VL	Visceral leishmaniasis
CL	Cutaneous leishmaniasis
MCL	Mucocutaneous leishmaniasis
PKDL	Post-kala azar dermal leishmaniasis
MMP	Mitochondrial membrane potential
HIV	Human immunodeficiency virus
WHO	World Health Organisation
